# Fimbrin phosphorylation by metaphase Cdk1 regulates actin cable dynamics in budding yeast

**DOI:** 10.1038/ncomms11265

**Published:** 2016-04-12

**Authors:** Yansong Miao, Xuemei Han, Liangzhen Zheng, Ying Xie, Yuguang Mu, John R. Yates, David G. Drubin

**Affiliations:** 1 Department of Molecular and Cell Biology, University of California, Berkeley, California 94720-3202, USA; 2 School of Biological Sciences, Nanyang Technological University, Singapore 637551, Singapore; 3 School of Chemical and Biomedical Engineering, Nanyang Technological University, Singapore 637459, Singapore; 4 Department of Chemical Physiology, The Scripps Research Institute, La Jolla, California 92037, USA

## Abstract

Actin cables, composed of actin filament bundles nucleated by formins, mediate intracellular transport for cell polarity establishment and maintenance. We previously observed that metaphase cells preferentially promote actin cable assembly through cyclin-dependent kinase 1 (Cdk1) activity. However, the relevant metaphase Cdk1 targets were not known. Here we show that the highly conserved actin filament crosslinking protein fimbrin is a critical Cdk1 target for actin cable assembly regulation in budding yeast. Fimbrin is specifically phosphorylated on threonine 103 by the metaphase cyclin–Cdk1 complex, *in vivo* and *in vitro*. On the basis of conformational simulations, we suggest that this phosphorylation stabilizes fimbrin's N-terminal domain, and modulates actin filament binding to regulate actin cable assembly and stability in cells. Overall, this work identifies fimbrin as a key target for cell cycle regulation of actin cable assembly in budding yeast, and suggests an underlying mechanism.

## Abstract (web summary)

Metaphase cells preferentially promote actin cable assembly through cyclin-dependent kinase 1 (Cdk1) activity. Here the authors identify fimbrin as one of the main metaphase Cdk1 targets for cell cycle regulation of actin cable assembly in budding yeast.

## Graphical abstract

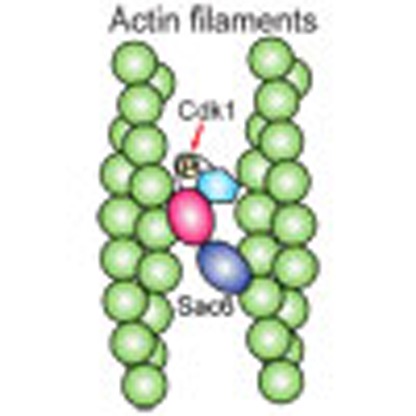

Actin filament networks are characterized by highly dynamic assembly and disassembly^1^,^2^. Specific proteins crosslink actin filaments (F-actin) into networks with distinct architectures. Different crosslinking patterns generate morphologically distinct actin networks that provide mechanical stability and force-generating capacity for cellular processes such as endocytosis, vesicle transport, cell motility and adhesion^1^,^2^,^3^. Filament-bundling proteins are either dimers or they contain multiple acting-binding domains, such as the calponin homology domain, gelsolin domain or the spectrin domain, and crosslink filaments into tightly packed bundles. Mammals have at least 13 classes of F-actin-bundling proteins that form distinct cellular structures and are regulated by different signalling pathways^3^. During cell growth or response to environmental changes, filament-bundling proteins are highly regulated to couple signal transduction with intracellular structural changes of actin networks^3^,^4^. Regulation of F-actin-bundling proteins often involves modulation of their binding, mediated through interaction or competition with other actin-binding proteins (ABPs), or through signals including calcium, ionic strength and phosphorylation^5^,^6^,^7^,^8^,^9^,^10^,^11^,^12^,^13^. Phosphoregulation is an important post-translational modification to modulate the F-actin crosslinking activity of bundling proteins. The ubiquitously expressed mammalian actin crosslinker, α-actinin, is regulated by tyrosine phosphorylation to either inhibit or stimulate actin-binding abilities, and therefore regulate the formation of stress fibres and maturation of focal adhesions^14^,^15^,^16^. Also, the neuronal crosslinking protein drebrin is phosphorylated by cyclin-dependent kinase 5 at Ser142 to regulate F-actin bundling in growth cone filopodia^11^.

Fimbrin (sometimes called plastin) is a highly conserved F-actin-bundling protein present from yeast to mammals. Mammalian L-plastin is homologous to budding yeast fimbrin (Sac6) and can substitute for yeast fimbrin *in vivo*^17^. Sac6 is the major F-actin crosslinking protein in budding yeast, while fission yeast has both an α-actinin-like protein (Ain1) and a fimbrin (Fim1) that are important for actin functions^18^. Fimbrins are generally characterized by an N-terminal headpiece domain, containing a calcium-binding domain (EF hand domain), and two C-terminal actin-binding domains (ABD1 and ABD2). The EF hand domain is separated from ABD1 by a disordered loop region. Calcium binding induces a conformational change in L-plastin that negatively regulates its actin-binding activity^19^,^20^. In contrast, cyclic AMP-dependent protein kinase phosphorylates Ser5 of L-plastin on the headpiece domain and positively regulates immune synapse formation and actin filament dynamics. L-plastin phosphorylation on Ser5 increases F-actin binding, aids targeting of L-plastin to focal adhesions and lowers the dissociation rate of L-plastin from actin filaments *in vivo*^21^,^22^. As in most eukaryotes, actin filaments undergo marked changes in organization and assembly through the cell cycle. However, how phosphorylation might contribute to this regulation, and the identity of the key targets, is largely unclear.

Budding yeast contain two formins, Bni1 and Bnr1, to initiate actin cable assembly^2^,^23^. While Bnr1 appears to localize exclusively at the bud neck, the Bni1 localization changes gradually throughout the cell cycle. In late G1, it localizes at the tip of the growing bud, its localization becomes diffuse from S-phase through metaphase, and then it concentrates at the bud neck in late anaphase^23^. Changes of Bni1 localization are thought to play essential roles in regulating actin cable assembly and organization. Throughout the cell cycle, actin cables are assembled by collaborative functions of Bni1 and other ABPs, including tropomyosin for filament stabilization, Sac6 for filament bundling, type V myosin for tuning actin cable motility and depolymerization factors^23^,^24^,^25^. Global analysis of cyclin-dependent kinase 1 (Cdk1) substrates revealed that multiple ABPs, including Bni1, Crn1, Bud6, Abp1, Myo2, Bud14 and Hof1 are dynamically phosphorylated by Cdk1 at different cell cycle stages^23^,^26^. Interestingly, >90% of Cdk1 phosphorylation sites are present in intrinsically disordered regions (IDRs), which are highly enriched among ABPs^26^,^27^. IDRs disrupt or enhance protein–protein interactions^26^,^27^. Phosphorylation is an essential mechanism for switching the protein stability and interactions of IDRs^28^. However, how Cdk1 regulates ABPs via IDRs during actin cable assembly remains to be determined.

We previously found that actin cable assembly is regulated through the cell cycle via Cdk1 activity. High expression of the cyclin B Clb2–Cdk1 complex promotes actin cable assembly during metaphase^20^. However, several important questions about Cdk1 function in actin assembly regulation remain unanswered, such as which ABPs are phosphorylated by the Clb2–Cdk1 complex for actin cable assembly regulation and how does phosphorylation tune their interaction with actin filaments. Previously, assembly of actin cables in cell extracts identified ABPs associated with cables, which are candidates to be cell cycle regulation targets^29^. Here we identify Sac6 as an important metaphase Cdk1 target. Sac6 is phosphorylated by the metaphase Clb2–Cdk1 complex at the N-terminal disordered region between the EF hand domain and ABD1. Cdk1 phosphorylation is predicted to induce a conformational change in the disordered loop region, and to thus facilitate Sac6 binding to actin filaments.

## Results

### Sac6 is phosphorylated by the mitotic cyclin–Cdk1 complex

**Figure 1:**
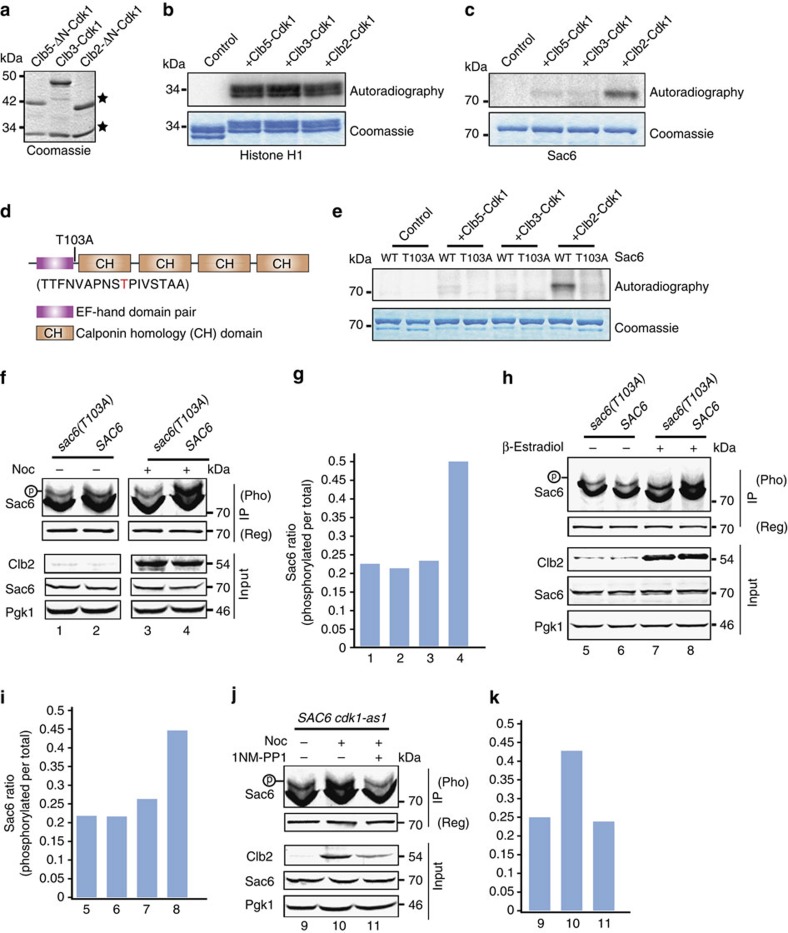
Phosphorylation of fimbrin (Sac6) by Cdk1. (**a**) Purification of cyclin–Cdk1 complexes from yeast. Coomassie blue staining shows Clb5–ΔN–Cdk1, Clb3–Cdk1 and Clb2–ΔN–Cdk1 purifed from yeast. (**b**) Normalization of kinase activity using histone H1. An amount of 2 μg of histone H1 was incubated with 12 ng of Clb5–ΔN–Cdk1, 11 ng of Clb3–Cdk1 and 6.5 ng of Clb2–ΔN–Cdk1 in the presence of [γ^32^P]-ATP at room temperature (25 °C) for 20 min. (**c**) Sac6 phosphoryaltion using same H1 kinase activities of cyclin–Cdk1 complexes. An amount of 1 μg of Sac6 protein was incubated with 6 ng Clb5–ΔN–Cdk1, 5.5 ng of Clb3–Cdk1 and 3.25 ng of Clb2–ΔN–Cdk1 in the presence of [γ^32^P]-ATP at room temperature for 20 min. (**d**) Identification of Sac6 residue T103 as a Clb2–Cdk1 target using mass spectrometry. (**e**) Clb2–ΔN–Cdk1 phosphoryaltes WT Sac6 but not Sac6-T103A. An amount of 1 μg Sac6 or Sac6-T103A was incubated with different cyclin–Cdk1 complexes. Phosphorylation was analysed by autoradiography following SDS–PAGE in **b**,**c** and **e**. (**f**,**g**) Asynchronous and nocodazole arrested *SAC6* and *sac6(T103A)* cells were collected, respectively, and subjected to immunoprecipitation (IP). Proteins were resolved on either regular or phosphate-affinity (Phos-tag), and blotted using Sac6, metaphase cyclin Clb2 and loading control Pgk1 antibodies. Numbers in **g** correspond to lane numbers in **f**. (**h**,**i**) *SAC6* and *sac6(T103A)* cells, carrying ADH1pr-GAL4BD-hER-VP16 (GEV), were induced with 100 nM of β-estradiol, collected and subjected to IP. (**j**,**k**) *SAC6* cells were collected from asynchronous and nocodazole arrested cells. Nocodazole arrested cells were treated with or without additional 1-NM-PP1 before being subjected to IP. The intensity ratio of phospholyated Sac6 species to total Sac6 in **f**,**h** and **j** (upppest panel, Phos-tag gel) was analysed and is shown in the graphs **g**,**i** and **k**, respectively. Symbol (p) indicates the phosphorlyated species of Sac6 on phos-tag gel in **f**,**g**,**i** and **k**.

Each cell cycle stage is established by a different cyclin-dependent kinase complex^30^. To determine whether specific cyclin–Cdk1 complexes can phosphorylate ABPs to regulate actin cables during metaphase, we first performed a radioactivity-based kinase assay on purified ABPs incubated with different cyclin–Cdk1 complexes. We tested whether Cdk1 can phosphorylate Act1 and several ABPs, including Tpm1, Pfy1, Cof1, Cap2, Abp1, the Bni1 formin C terminus (FH1-COOH), Crn1 (1–600 aa) and Sac6, which were identified previously by mass spectrometry as cable-associated proteins^29^. We found that the yeast fimbrin Sac6 is a specific Clb2–Cdk1 substrate. Bni1 and Crn1 were also be phosphorylated by cyclin–Cdk1 complexes (Supplementary Fig. 1a,b), while actin (Act1), tropomyosin (Tpm1), profilin (Pfy1), cofilin (Cof1) and capping protein (Cap2) were not phosphorylated (Supplementary Fig. 1c). In this study, we focused on Cdk1 phosphoregulation of Sac6. Sac6 was incubated with Cdk1 complexes containing the S-phase triggering cyclin Clb5 (Clb5–ΔN–Cdk1), the G2/M cyclin Clb3 (Clb3–Cdk1) and the mitotic cyclin Clb2 (Clb2–ΔN–Cdk1). We first purified the three cyclin–Cdk1 complexes expressed under control of the GAL promoter in budding yeast (Fig. 1a). Because it has been previously shown that cyclin–Cdk1 complexes have different intrinsic kinase activities^31^, we normalized kinase activity of the different cyclin–Cdk1 complexes with a commonly used model substrate, histone H1 (Fig. 1b). We also purified polyhistidine-tagged full-length Sac6 protein (70 kDa) from yeast. Phosphorylation of full-length Sac6 was assayed in the presence of equal active units of Clb5–ΔN–Cdk1, Clb3–Cdk1 and Clb2–ΔN–Cdk1 complexes. Interestingly, we found that Sac6 is specifically phosphorylated by the Clb2–Cdk1 complex and not by Clb5–Cdk1 and Clb3–Cdk1 complexes (Fig. 1c).

Sac6 contains three minimal Cdk1 (Ser/Thr–Pro (S/T–P)) consensus motifs (T103, T366 and T589) as potential phosphorylation sites for cyclin–Cdk1 complexes. To investigate how metaphase Cdk1 regulates Sac6, we first set out to map Clb2–Cdk1 phosphorylation sites on Sac6. We incubated 5 μg of purified yeast Sac6 with or without the Clb2–Cdk1 complex, and subjected the Sac6 to mass spectrometry-based phosphorylation site mapping. We found that Sac6 purified from yeast without kinase treatment already has a basal phosphorylation on one of its Cdk1 consensus phosphorylation S/T–P motifs at T103 (Fig. 1d). T103 resides in an unstructured loop region between the EF hand and ABD1 domains. Incubation of Clb2–Cdk1 with Sac6 resulted in around a twofold spectrum count enrichment for T103-containing peptides (Supplementary Data 1). However, the other two potential Cdk1 sites (T366 and T589) were not identified as phosphorylation sites by mass spectrometry. To confirm that the T103 identified by mass spectrometry is the sole phosphorylation site for the Clb2–Cdk1 complex, we replaced threonine 103 with alanine (Sac6-T103A) by site-directed mutagenesis. We next purified Sac6-T103A from yeast and performed a kinase assay in parallel with wild-type (WT) Sac6 using the Clb5–ΔN–Cdk1, Clb3–Cdk1 and Clb2–ΔN–Cdk1 complexes. We found that mutation of T103 to alanine completely abolished Sac6 phosphorylation by Clb2–Cdk1 (Fig. 1e).

We next asked whether Clb2–Cdk1 phosphorylates Sac6 *in vivo* during metaphase. We first generated yeast cells expressing Sac6-T103A under its endogenous promoter. Then, using anti-Sac6 antibodies, we performed immunoprecipitations from asynchronous *Sac6* and *sac6(T103A)* cells, and cells arrested in metaphase by 3 h pretreatment with 15 μM nocodazole, which is widely used to achieve a metaphase arrest. Immunoprecipitated proteins were resolved on phos-tag gels and by conventional SDS–polyacrylamide gel electrophoresis (SDS–PAGE) to assess phosphorylation. We found that metaphase cells, which contain elevated Clb2, show a higher level of Sac6 phosphorylation than was observed in asynchronous cells (Fig. 1f,g). In contrast, no difference in Sac6-T103A phosphorylation was observed between metaphase and asynchronous cells (Fig. 1f,g). This result indicates that T103A is a specific Sac6 phosphorylation site during metaphase *in vivo*. Intriguingly, we also noticed that both Sac6 WT and Sac6-T103A have a basal level of phosphorylation *in vivo*, suggesting that Sac6 is also phosphorylated by another kinase. Next, we asked whether elevated Sac6 phosphorylation at metaphase is via the Clb2–Cdk1 complex. *CLB2* transcription under control of the GAL promoter was activated on addition of 100 nM of β-estradiol. Clb2 levels showed an approximately eightfold increase after 1 h of β-estradiol addition. Clb2 overexpression caused higher levels of phosphorylation for WT Sac6, but not for Sac6-T103A (Fig. 1h,i), agreeing with the results for the metaphase-arrested cells (Fig. 1f). Only the level of Sac6 phosphorylation, not Sac6 expression, was induced by overexpressing Clb2 (Fig. 1h). In addition, we further tested whether Sac6 phosphorylation is directly and specifically regulated by Cdk1 kinase activity at metaphase. To address this question, we replaced endogenous Cdk1 with an analogue-sensitive allele (*cdk1-as1*), in which the kinase activity can be rapidly and specifically inhibited by the small molecule 1-NM-PP1 (refs 26, 32, 33). *cdk1-as1* cells were synchronized at metaphase by nocodazole, and then treated with 20 μM of 1-NM-PP1 for 30 min. Inhibition of Cdk1 activity caused a clear decrease of Sac6 phosphorylation in metaphase-arrested cells (Fig. 1j,k). Taken together, the above *in vitro* and *in vivo* phosphorylation results provide evidence that Sac6 is specifically phosphorylated on T103 during metaphase by Clb2–Cdk1.

### Cdk1 phosphorylates Sac6 to regulate actin cable dynamics

**Figure 2:**
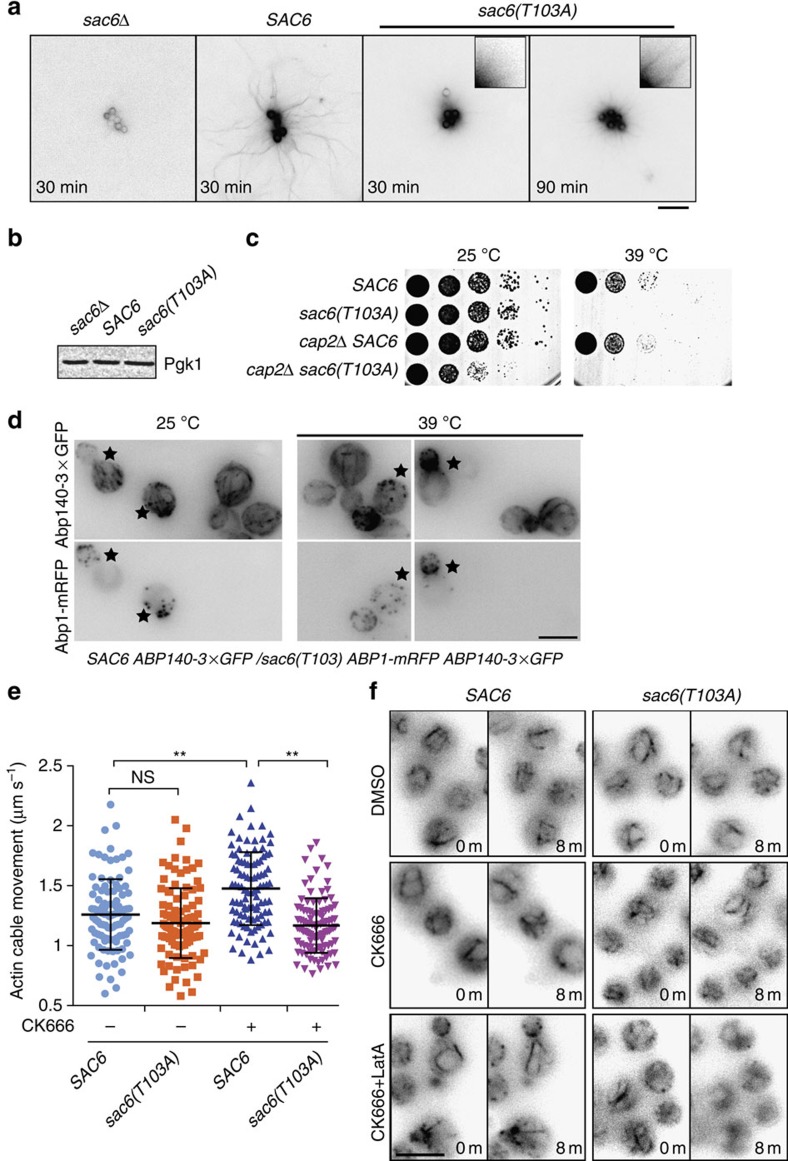
Sac6 phosphorylation regulates actin cable dynamics and is important for normal cell growth. (**a**) GST-Bni1 FH1-COOH-functionalized beads were added to cytoplasmic extracts from hydroxyurea (HU)-arrested yeast cells of indicated genotypes expressing Abp140-3 × GFP. (**b**) Protein concentration for cytoplasmic extracts used for actin reconstitution in **a** is compared using Pgk1 by immunoblotting. (**c**) Growth test by serial dilution of WT, *cap2Δ*, *sac6Δ*, *cap2Δ SAC6*, *cap2Δ sac6(T103A)* double mutant on YPD plates. Plates were incubated at indicated temperature for 24 h. (**d**) Fluorescent pattern of actin filaments in *SAC6* and *sac6(T103A)* showed by maximum intensity Z-projection. Cells were grown at 39 °C for 30 min before imaging. Star indicates *sac6(T103A)*. (**e**) Actin cable movement speed in HU-arrested cells treated with or without CK666 (*n*=100). A concentration of 50 μM CK666 treatment was applied for 30 min at room temperature. Actin filament elongation rate was measured by monitoring the ends of the elongating cables. (**f**) Depolymerization of actin cables by Lat A treatment. Real-time imaging of actin cables at the cell cortex in *SAC6* and *sac6(T103A)* cells. Cells were pretreated with 50 μM CK666 or DMSO for 30 min at room temperature before adding 0.4 μM Lat A. Images were acquired at 2-min intervals after adding Lat A. Images at time point 0 and 8 min are shown. Scale bars, 5 μm. Error bars are in s.d. One-way analysis of variance was used. **P<0.0001. DMSO, dimethylsulphoxide; NS, not significant.

We next asked how phosphorylation of Sac6 at T103 affects actin cable assembly and cell growth. We reconstituted actin assembly *in vitro* using cell extracts made from *sac6Δ*, *SAC6* and *sac6(T103A)* cells, all expressing 3 × green fluorescent protein-tagged Abp140 (Abp140-3 × GFP) as an actin cable marker. Using a reconstitution assay we employed previously^29^, cells were first arrested at metaphase using hydroxyurea, and then lysed for cytoplasmic extract preparation. Polystyrene beads coated with Bni1 FH1-COOH were incubated with extracts for 30 min before imaging. We found that actin cables were unable to assemble in extracts from *sac6Δ* mutants, but assembled robustly in WT extracts (Fig. 2a). In *sac6(T103A)* cell extracts, short actin filaments pointing out from the beads assembled, but there were no clear actin cable structures (Fig. 2a). Extension of the incubation time to 90 min did not significantly increase actin cable formation (Fig. 2a). All of the beads in each assay showed similar patterns of actin cable assembly. Although it is difficult to identify the ends of individual actin filaments, and to precisely measure their lengths, we could detect the ends of actin cables ∼20 μm from the beads (*n*=153) after 30 min in *SAC6* extracts. However, the ends of actin cables in *sac6(T103A)* extracts were only ∼6 μm from the beads (*n*=181; Fig. 2a). Observed differences in actin assembly were not caused by changes in Sac6 levels in different strains used (Fig. 2b; Supplementary Fig. 2a).

We next asked how Sac6 phosphorylation at T103 affects cell growth and actin structures. We found that *sac6(T103A)* does not show a clear growth defect at 25 °C, but elevating the temperature to 39 °C causes lethality in *sac6(T103A)* (Fig. 2c). Growth at 37 °C revealed a mild growth defect (Supplementary Fig. 2b). The *sac6Δ* allele and the *sac6-102* point mutant, which has a mutation of serine to phenylalanine at residue 423, within the ABD2 domain, exhibited similar growth defects^34^ (Supplementary Fig. 3a). To test Sac6-T103A function in a more sensitive way at 25 °C, we further perturbed actin filament assembly by deleting the gene encoding the actin capping protein subunit Cap2. Absence of Cap2 showed a strong synthetic sickness at 25 °C and total lethality at 39 °C with *sac6(T103A)* (Fig. 2c), indicating that Sac6 function is impaired by a phospho-defective mutation at T103, while *sac6-102* has stronger genetic interaction with *cap2Δ* and showed synthetic lethality (Supplementary Fig. 3b). We next tested the importance of Sac6 phosphoregulation in the context of impaired formin and myosin V function, since these proteins are key actin cable assembly regulators^24^. *sac6(T103A)* showed a clear growth defect in combination with *bni1Δ* but not with *bnr1Δ* or *myo2-16* (Supplementary Fig. 4a). Actin cable morphology and assembly were also defective when *sac6(T103A)* was combined with *bni1Δ*, effects that were not seen in combination with *bnr1Δ* or *myo2-16* (Supplementary Fig. 4b–d). Interestingly, *sac6(T103A)* cells showed faster cable elongation than *SAC6* cells in both the *bni1Δ* and *myo2-16*, but not in the *bnr1Δ* background (Supplementary Fig. 4e). We also tested whether Abp140-3 × GFP behaves similar to Abp140-GFP, which is generally used for measuring actin cable dynamics^24^. We found that Abp140-GFP and Abp140-3 × GFP showed similar cable organization and average cable elongation speeds, although the proportion of cables with velocities >2 μm s^−1^ was slightly more (∼10%) for Abp140-GFP than for Abp140-3 × GFP (Supplementary Fig. 4f,g).

We next investigated how Sac6 phosphoregulation impacts actin cable dynamics *in vivo*. We examined actin cables in *SAC6* and *sac6(T103A)* cells expressing Abp140-3 × GFP. To minimize the fluorescent signal variation between the samples, we mixed both the cell types together on the same slides. To distinguish *SAC6* from *sac6(T103A)* cells, an additional red fluorescent marker, Abp1-mRFP, which associates with actin patches, was expressed in *sac6(T103A)* cells. We found that actin cables disappeared in *sac6(T103A)* cells in response to incubation for 30 min at 39 °C, while *sac6(T103A)* and *SAC6* cells showed similar actin cable patterns at 25 °C (Fig. 2d). The actin cable defect observed at 39 °C (Fig. 2d) was not caused by a difference in the Sac6 protein levels (Supplementary Fig. 3c). The defect of actin cable in *sac6(T103A)* enhanced gradually by the increasing of temperature (Supplementary Fig. 2b). To further test the phosphorylation effects, we also generated phosphomimetic *sac6(T103E)*. However, *sac6(T103E)* also showed the actin cable defect at elevated temperature (Supplementary Fig. 2c), indicating a disability in functional mimic of phosphorylated T103, which is commonly observed when replacing ‘S' or ‘T' by ‘D' or ‘E'^35^. Therefore, we only performed functional comparison between *SAC6* and *sac6(T103A)* in this report.

Next, we set out to determine how Sac6 phosphorylation affects actin cable dynamics in living cells. We first arrested the cells in metaphase by hydroxyurea addition at 25 °C to induce elevated Sac6 phosphorylation. While *sac6(T103A)* and *SAC6* showed the similar patterns of polarized actin patches, *sac6(T103A)* showed a mild defect in actin cable assembly compared with *SAC6* (Supplementary Fig. 5). However, when imaging Abp140-3 × GFP and following the positions of cable ends over time near the cell cortex^24^, we still observed comparable speeds of actin cable movement in *sac6(T103A)* (1.20±0.30 μm s^−1^; ±s.d.) and in *SAC6* (1.28±0.30 μm s^−1^) cells (Fig. 2e).

Sac6 functions both in the branched actin networks of actin patches and in unbranched actin cables. To investigate in greater depth how Sac6 phosphorylation regulates dynamics of unbranched actin cables, we specifically depleted the actin filament population in actin patches using the Arp2/3 inhibitor CK666 (ref. 36). First, we performed a dose–response for CK666 to identify the minimum concentration that can effectively abolish actin filament assembly in actin patches. Using Abp1-mRFP as an indicator for *in vivo* actin assembly at actin patches, we found that 50 μM CK666 treatment at 25 °C largely diminished the Abp1 signal within 30 min (Supplementary Fig. 6a), but did not result in a detectable defect in actin cables (Supplementary Fig. 6b). We also found that in response to the CK666 treatment, actin cable velocity was significantly increased to 1.50±0.31 μm s^−1^ in *SAC6* cells (Fig. 2e), likely the result of an elevated pool of unassembled actin. However, an identical treatment in *sac6(T103A)* cells did not lead to a significant increase of actin cable velocity (1.18±0.23 μm s^−1^; Fig. 2e), indicating an inability to increase the actin cable assembly in response to an increased actin monomer pool.

Next, we tested whether Sac6 phosphorylation affects actin filament depolymerization dynamics by treating cells with the actin monomer-binding drug Latrunculin A (Lat A). Lat A in the 100–200 μM range, causes fast actin filament depolymerization in actin patches, and almost immediate disappearance of actin cables. We titrated the Lat A to find a concentration that would enable us to follow the dynamics of actin cable depolymerization by fluorescence microscopy *in vivo*. We found that 0.4 μM Lat A allowed us to follow actin cable disappearance over a time period of 15 min. To test how *sac6(T103A)* affects actin cable depolymerization, we pretreated *SAC6* and *sac6(T103A)* cells with 50 μM CK666 for 30 min before addition of 0.4 μM Lat A. Actin cables were largely depolymerized after 8 min of Lat A treatment in *sac6(T103A)* cells, while much less of an actin cable depolymerization effect was observed in *SAC6* cells (Fig. 2f; Supplementary Fig. 7). Actin cables were still present in *SAC6* cells after 15 min of treatment (Supplementary Fig. 7). These Lat A results establish that in the absence of Sac6 phosphorylation by Cdk1, actin cable formation and stability are perturbed.

### T103A phosphorylation site mutant impairs Sac6 localization

Several factors, including Cof1, Sac6 and Cap1/2, regulate both actin cables and actin patches. Most of these proteins are observed to predominantly localize to patches in live-cell imaging^23^,^37^. We asked whether a phosphorylation site mutant of Sac6 affects its subcellular localization. Both actin patch and cytosolic signals were observed for Sac6-GFP and Sac6-T103A-GFP. We determined the ratio of fluorescent signals in patches versus the cytoplasm. We found that Sac6-T103A-GFP has a reduced ratio of patch/cytosol localization compared with Sac6-GFP (3.30±1.04 versus 1.52±0.35; Supplementary Fig. 6c,d). We next measured the life time at endocytic patches of Abp1-mRFP^29^ in *SAC6* and *sac6(T103A)* cells. At 25 °C, the Abp1-mRFP life time was similar in WT and mutant cells (9.6±1.4 versus 9.8±1.3; Supplementary Fig. 6e,f). However, at a higher temperature (39 °C), Abp1 life time was shorter in *SAC6* cells (7.1±1.1), indicating faster endocytosis, but not appreciably in *sac6(T103A)* (9.0±2.0; Supplementary Fig. 6e,f).

### T103A mutant impairs Sac6 binding to yeast actin *in vitro*

**Figure 3:**
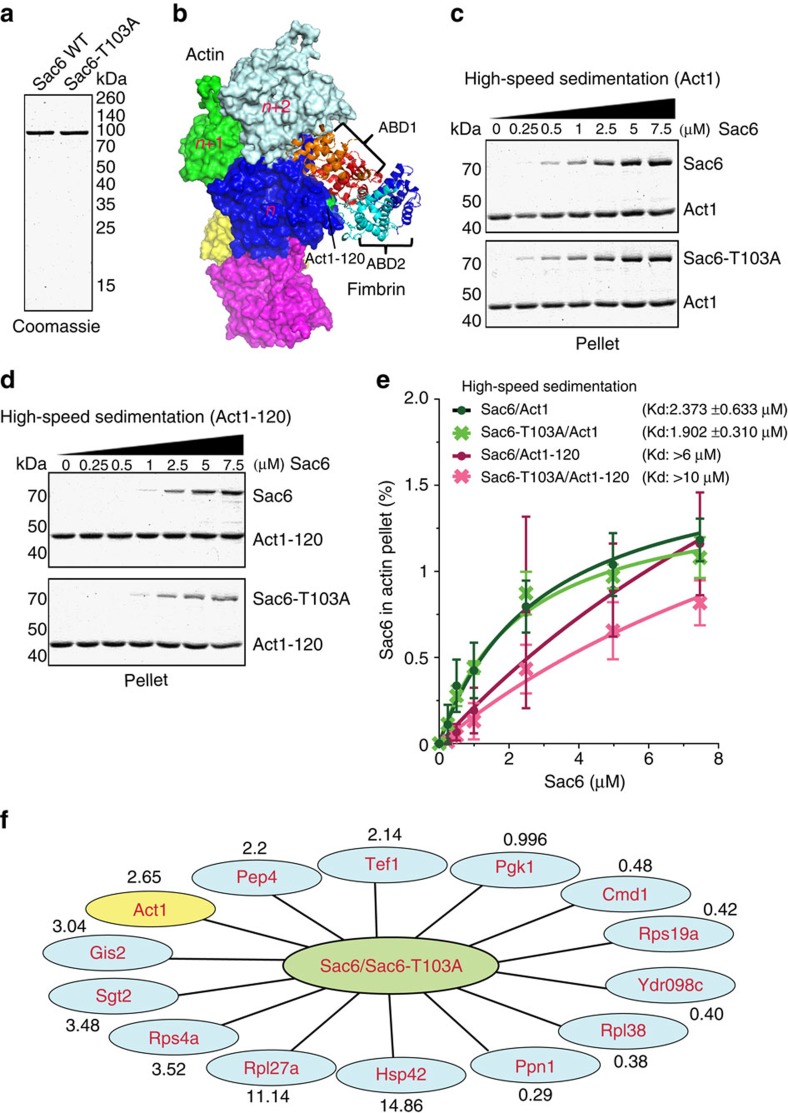
Phospho-mutation of Sac6 attenuates its yeast F-actin-binding capacity and other interaction partners. (**a**) Coomassie blue-stained gels of yeast-purified Sac6 WT and Sac6-T103A. (**b**) Structural model of actin filament (PDB ID: 3J8I)^69^ and fimbrin proteins (PDB ID: 1RT8)^70^ using PyMOL. Five actin monomers coloured white, green, blue, yellow and purple, respectively, are shown in the F-actin structure. Corresponding mutated residues for Act1-120 (E99A, E100A) are indicated in green on the actin monomer ‘n'. Four calponin homology (CH) domains, CH1–CH4, in fimbrin's actin crosslinking core ABD1 and ABD2 are shown in red, orange, cyan and blue, respectively. (**c**,**d**) Coomassie blue-stained gels of actin filament pellets after a range of Sac6 and Sac6-T103A concentrations were added. F-actin was assembled from 1.25 μM Mg-ATP-yeast actin, incubated with Sac6 or Sac6-T103A, and spun at 100,000*g* for 20 min. Molecular weight is indicated on the left. (**e**) Plot of the amount of (•) Sac6 WT and (**X**) Sac6-T103A pelleted with Act1 (green) and Act1-120 (magenta), respectively, over a range of fimbrin concentrations. Curve fits revealed an equilibrium dissociation constant of Sac6 variants (•, green) 2.373±0.633 μM (s.e.) for Sac6/Act1 (*n*=3), (**X**, green) 1.902±0.310 μM (s.e.) for Sac6-T103A/Act1 (*n*=3), (•, magenta) >6 μM for Sac6/Act1-120 (*n*=4) and (**X**, green) >10 μM for Sac6-T103A/Act1-120 (*n*=4). (**f**) Interaction partners of Sac6 and Sac6-T103 were identified by SILAC coupled mass spectrometry and displayed using Cytoscape. The data were normalized to Sac6 protein. Differences in ratios measured for the binding partners of Sac6 and Sac6-T103A are indicated by numbers.

We next sought to test whether Sac6 phosphorylation affects its interaction with actin filaments. We used a high-speed co-sedimentation assay to test the binding activity of different combinations of yeast actin and Sac6 variants. We purified Sac6 WT and Sac6-T103A and tested their interactions with actin filaments polymerized from yeast Act1 and Act1-120 protein (Fig. 3a; Supplementary Fig. 6g). A near-atomic resolution structural model for the actin crosslinking core of fimbrin bound to F-actin was derived from structural alignment and docking (Fig. 3b; Supplementary Methods). Act1-120 has E99A and E100A mutations on its interaction interface with Sac6 (Fig. 3b; Supplementary Fig. 8a)^38^. In agreement with previous reports, Act1-120 had a lower affinity for Sac6 than WT actin (Fig. 3c,d)^38^. While WT actin did not show clear binding differences towards Sac6 and Sac6-T103A (Fig. 3c,e), Act1-120 showed a pronounced reduction in binding to Sac6-T103A compared with Sac6 (Fig. 3c–e).

### T103A mutant reduces Sac6 interaction with actin *in vivo*

Phosphorylation of the fimbrin/plastin headpiece has been suggested to regulate its affinity for actin and its other binding partners^39^. We investigated how the affinity of Sac6 towards its binding partners is influenced by Cdk1 phosphorylation *in vivo*. We employed a quantitative proteomic approach using stable isotope labelling with amino acids in cell culture (SILAC) coupled with Multi-Dimensional Protein Identification Technology^40^. Tandem affinity purification (TAP) was used to isolate Sac6 variants and their associated binding partners. *SAC6-TAP* and *sac6(T103A)-TAP* cells were grown in yeast extract peptone dextrose (YPD) media containing natural (‘light') amino acids or ‘heavy' isotope amino acids, respectively. Equal quantities of soluble proteins were obtained from *SAC6-TAP* and *sac6(T103A)-TAP* cells separately, and were subsequently combined for TAP tag purification^26^,^41^. Thirteen interaction partners of Sac6-TAP and Sac6-T103A-TAP with more than twofold binding difference were identified by quantitative mass spectrometry (Fig. 3f; Supplementary Data 2). As a protein control, phosphoglycerate kinase 1 (PGK1) showed a ratio of 0.996 (Fig. 3f), which indicates an equal presence as a contaminant in both Sac6 and Sac6-T103 samples, thus giving confidence in any protein found to be preferentially associated with one form of Sac6 over the other. Interestingly, actin (Act1) showed 2.65 enrichment with Sac6 WT relative to Sac6-T103A (Fig. 3f). In addition, we employed an immuno-isolation approach, which also showed reduced actin association for Sac6-T103A compared with WT Sac6 (Supplementary Fig. 6h). Taken together, the above *in vitro* and *in vivo* results indicate that Sac6 phosphorylation increases its interaction with actin.

### Sac6 phosphorylation stabilizes a loop containing T103

T103 is located in a disordered loop of Sac6, from 90 aa to 140 aa, connecting the EF hand domain to ABD1. At the N terminus of the T103-containing loop, the EF hand domain is known to face away from the actin filament that interacts with ABD1, and to have a constrained orientation with respect to the EF hand helices^42^,^43^,^44^. At the C terminus of the loop containing T103, ABD1 and ABD2 form a crossbridge between actin filaments with limited flexibility. Disordered regions are often found to be the flexible linkers (or loops) for connecting adjacent domains. They also are often targets for phosphorylation, which can change local protein conformation and function^45^. To test whether T103 phosphorylation might change the conformation of the disordered region in which it resides, we performed a conformational simulation using the metadynamics approach^46^. How phosphorylation can drive conformational changes of peptides has been successfully modelled *in silico*^47^.

**Figure 4:**
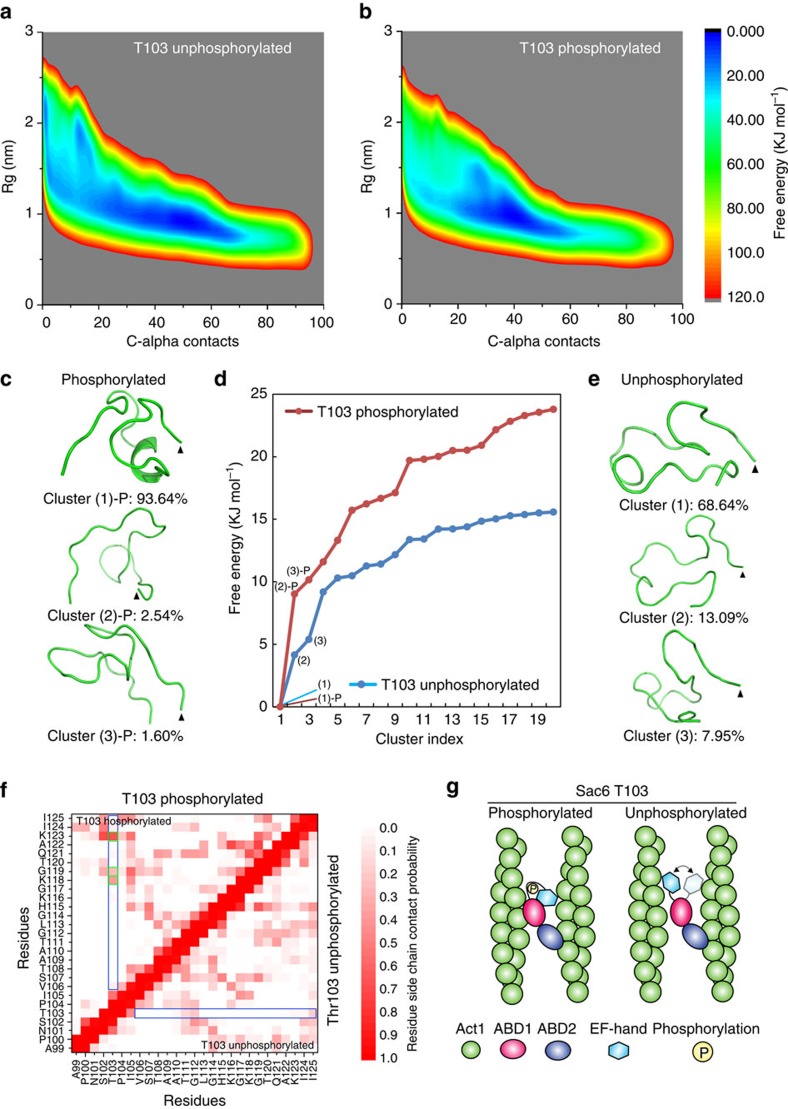
MetaDynamics simulation of Sac6 loop region with or without T103 phosphorylation. The free energy surfaces plotted based on two CVs (Rg and C-alpha contact number) for Sac6 N-terminal peptide between the EF hand and ABD1 domain without (**a**) and with T103 phosphorylation (**b**). The colour key indicates the scale of free energy values in units of kJ mol^−1^. (**c**,**e**) The representative structures of the three lowest-free-energy clusters. C terminus is indicated by an arrowhead. (**d**) Free energies of the first 20 lowest-free-energy clusters after reweighting. (**f**) Contact probability map of the residue side chains in simulated peptides. The interaction possibilities for the residue side chains within T103-phosphorylated and -unphosphorylated peptides are shown in the upper-left and lower-right triangles, respectively. Colour key indicates the range of the interaction probabilities. Blue boxes indicate the positions of phosphorylated and -unphosphorylated T103 (blue box), respectively. Green boxes indicate residues that interact with phosphorylated T103. (**g**) A model of the conformational changes of Sac6 by T103 phosphorylation. Compared with the unphosphorylated protein, T103 phosphorylation introduces higher stability to the N-terminal EF hand domain for better Sac6 interaction with actin filaments.

In our study, considering that phosphorylation changes the net charge of the loop containing T103, and that charge–charge interactions may play an important role in loop stability and conformation, the segment of 99–126 (APNSTPIVSTAATGLQHKGKGTQAKII), which includes three lysine residues, was chosen for a detailed conformational study. The length of this segment, 27 amino acids, is permissive for all-atom explicit solvent molecular dynamics simulation studies. The structure of the T103 peptide was simulated starting from a linear conformation in an aqueous environment at room temperature. Interestingly, the phosphorylated T103 peptide is predicted to have conformations with less flexibility compared with the unphosphorylated peptide. Free energy surface analysis showed that the peptide with unphosphorylated T103 is highly flexible with broad area of low free energy (Fig. 4a). In contrast, the peptide containing phosphorylated T103 showed a smaller low-free-energy zone (Fig. 4b), indicating that the flexibility of structural conformations was constrained. In addition, we verified the free-energy calculation using a reweighting method by clustering the peptide conformations^48^. T103 phosphorylation produced a stable conformation that occupied 93.64% of the total conformation probabilities (Fig. 4c,d; Supplementary Fig. 9a–g). The energy gap between the first and second cluster is around 10 kJ mol^−1^, indicating a large energy requirement for conformational switching. However, at least two dominant conformations were derived for peptides with unphosphorylated T103 that occupied 68.6% and 13.1% of the total population, respectively (Fig. 4d,e). The free energy difference between these two populations is <5 kJ mol^−1^, indicating easy conformational switching and high flexibility.

To next investigate the how other residues interact with T103 in the Sac6 IDR, we constructed a contact probability map for both phosphorylated and unphosphorylated T103 peptides. Phosphorylated T103 showed two highly intense coloured regions on the contact probability map, indicating stable interactions (Fig. 4f). We observed two types of interaction of the phosphate group on T103 with other residues, including K118, G119 and K123 (Fig. 4f). First, the phosphate group on T103 interacts with G119 by a hydrogen bond, which creates a turn conformation (Supplementary Fig. 9h). In addition, G119 also has a hydrophobic interaction with I105, which might further facilitate the restricted conformation of the bend at G119. Second, phosphorylated T103 interacts with the side chains of lysine residues (K118 and K123) and forms salt bridges (Fig. 4f; Supplementary Fig. 9h), which is a typical interaction for a phosphorylated residue^49^. Similarly, a recent study also showed that phosphorylation of Tau protein created a turn by a hydrogen bond between a phosphate group on T205 and an amide proton on G207, and a salt bridge between phosphorylated T205 and R209 (ref. 50). In contrast, the unphosphorylated T103 peptide only showed low contact probabilities with the side chains of other residues, and thus resulted a flexible peptide (Fig. 4f).

Taken together, our simulations predict that T103 phosphorylation will enhance stability of the N-terminal disordered region, which we propose will constrain flexibility of the EF hand domain in the vicinity of the ABD1 domain, which will facilitate interaction between ABD1 and actin filaments (Fig. 4g).

## Discussion

In essentially all eukaryotic cells types, the actin cytoskeleton is rearranged dynamically during mitosis. In vertebrate cells, stress fibres disassemble and actin filaments at the cell cortex are remodelled for cell rounding^51^. Cdk1 activity is required for mitotic cell rounding, because in its absence the cells flatten^52^. Yeast cells also undergo pronounced, programmed changes in actin organization throughout the cell cycle^53^. However, the mechanism that underlies actin organization changes throughout the cell cycle is not well understood in any cell type. Yeast cells are attractive for studies of cell cycle regulation of cytoskeleton assembly because the cell cycle is well studied and many mutants and reagents are available.

Some actin cytoskeleton targets of cell cycle regulation have been identified. The mammalian formin mDia3 is phosphorylated by Aurora B kinase to regulate metaphase chromosome alignment^54^. In addition, the fission yeast formin Cdc12 is phosphorylated by the septation initiation network kinase Sid2 to regulate cytokinetic ring assembly and cell division^55^. In budding yeast, the mitotic cyclin–Cdk1 phosphorylates diverse ABPs^26^. For example, the IQGAP family protein Iqg1, which is involved in actin filament assembly^56^, is targeted by Cdk1 for actomyosin ring assembly^57^. Nevertheless, our understanding of cell cycle actin regulation will not be complete until all of the functionally important Cdk1 cytoskeletal targets have been identified.

Here we identified the budding yeast fimbrin Sac6 as a biologically important Cdk1 target for cell cycle regulation of actin assembly. Sac6 is phosphorylated by Cdk1 directly and we provided evidence from *in vivo* and *in vitro* experiments that this phosphorylation plays a crucial role in cell cycle regulation of actin cable assembly. We demonstrated that phosphorylation of Sac6 residue T103 is tightly regulated by Cdk1 during metaphase. Moreover, Sac6 phosphorylation increased Sac6 association with actin *in vitro* and *in vivo*. Identification of Sac6 interaction partners by SILAC identified interacting proteins and provided evidence that some of their interactions with Sac6 may be regulated by Cdk1. Tef1, a translation elongation factor 1a essential for actin interaction and bundling^58^,^59^, preferentially associated with more highly phosphorylated Sac6. Future studies are required to determine how Tef1 activity might contribute to actin cable regulation.

Having provided strong cell biology and biochemical evidence that Sac6 is an important Cdk1 target for cell cycle regulation of actin cable assembly, it was important to determine what effects phosphorylation might have on Sac6 and its interaction with actin. Using conformational simulations, we tested for the effects on the disordered loop region that contains T103. This loop links the Sac6 EF hand and ABD1 domains. Previously it was established that the EF hand domain can modulate affinity of the adjacent ABD1 domain^43^,^44^. Calcium was identified as one of the important factors in regulation of fimbrin/plastin EF hand domains, reducing affinity for actin filaments^60^. Binding to actin by *Tetrahymena* fimbrin, which lacks the N-terminal EF hand domain, does not respond to calcium^61^. In this report, we analysed phosphoregulation of Sac6 by Cdk1 in the disordered region. A sequence alignment (Supplementary Fig. 8b) shows that the disordered region of fimbrin evolves rapidly, which is consistent with the characteristics of flexible disorder^62^. Flexible disordered regions that are intrinsically unstructured are often associated with signalling and regulation^62^. Interestingly, Cdk1 consensus sites ‘S/T–P' are highly enriched in the fimbrin disordered regions of diverse Ascomycota species, the largest phylum of fungi (Supplementary Fig. 8b,c). This observation suggests that Cdk1 phosphoregulation of fimbrin in this region is a conserved regulatory mechanism among Ascomycota species during evolution.

When Sac6 bundles adjacent actin filaments, the EF hand domain is thought to face away from the ABD1-bound actin filament and to imbed itself inside a crevice between the two calponin homology domains in ABD1 (Fig. 4f)^43^,^44^. Our simulations indicated the phosphorylation of T103 on Sac6 restrains the conformational freedom of the unstructured loop, which joins the EF hand to ABD1 (Fig. 4f). Restraining the conformational freedom of an otherwise flexible polypeptide chain will reduce the entropic cost for forming stable complexes between the N-terminal EF hand, ABD1 and F-actin (Fig. 4f). This scenario is well supported by our analysis of the interaction of Sac6 with actin *in vitro* and *in vivo* (Fig. 3).

Cortical actin meshwork remodelling during cell rounding at metaphase is proposed to be largely dependent on crosslinking proteins^63^. This proposed central role for crosslinking proteins suggests that their phosphoregulation by mitotic Cdk1 may also occur in mammals. In addition to fimbrin, coronin and the formin Bni1 were also identified as direct Cdk1 targets (Supplementary Fig. 1), which suggests that Cdk1 phosphoregulation of actin cable assembly involves several targets. Here we also showed that in the background of Bni1 and Myo2 mutants, actin cables move faster in *sac6(T103A)* cells than in *SAC6* cells. This result suggests that fast actin cable elongation might be controlled by Bni1 and Myo2, and buffered and balanced by bundling via Sac6. In addition, localization of Bni1 becomes more delocalized on the cell cortex during metaphase than in G1. Sac6 phosphorylation at metaphase might help maintain polarized bundles before actin cables are converted to a filament meshwork following metaphase. Further studies are required to test the biological relevance of these targets, and to further study the mechanisms responsible for cell cycle regulation of actin cytoskeleton assembly.

## Methods

### Yeast culture conditions and plasmids

Yeast strains, plasmids and primers used in this study are listed in Supplementary Tables 1–3, respectively. Genes were deleted by replacing the gene open reading frames with the *Candida glabrata LEU2*, *ClonNAT* or *KanMX* cassettes. Genomic C-terminal tagging was performed as previously described^29^,^64^. In brief, PCR products that contain a selectable marker and regions of homology upstream and downstream of the target gene open reading frame were transformed into yeast cells using lithium acetate-based method^64^. pFA6a-GFP(S65T)-kanMX or pFA6a-GFP(S65T)-His3MX6 were used as templates for PCR for fluorescent fusion tagging^29^. Transformants were selected on either YPD medium plates containing 200 μg ml^−1^ Geneticin or synthetic complete and dropout medium plates. All strains were grown at 30 °C in standard-rich media (YPD) or synthetic media supplemented with appropriate amino acids, unless otherwise specified. Plates were incubated for 2 days before scoring cell growth. Genome-integration constructs of *sac6(T103A)* and *sac6(T103E)* were generated from the integration vector pRS306 that contains genomic DNA-derived expression cassette of *SAC6* via a Quick Change Lightning site-directed mutagenesis kit (Stratagene).

### Protein purification

Cells carrying leucine-deficient plasmids of *P*_*GAL*_*-SAC6-Strep-9xHis* or *P*_*GAL*_*-SAC6(T103)-Strep-9xHis* variants were grown in leucine-dropout media containing 2% raffinose to OD_600_ of ∼2 at 30 °C. The leucine-deficient plasmid has the advantage of weaker expression than WT *LEU2*, which selects for high-copy levels of the Sac6 variant-containing plasmids in cells^65^. GST-Bni1 FH1-COOH, Cap2 and Tpm1 were expressed and purified via 2-μm plasmid-based galactose-inducible system as previously described^29^. In brief, 1 × YP (yeast extract-peptone) and 2% galactose were added to the cells at OD_600_ of ∼2 for induction. Cells were grown for an additional 12 h at 30 °C, collected, resuspended in 20% volume of water and frozen in liquid N_2_. Frozen yeast tablets were ground into powder using a Freezer/Mill 6870 (SPEX SamplePrep). An amount of 5 g of yeast powder was thawed in 10 ml of lysis buffer (50 mM HEPES (pH 7.5), 500 mM KCl, 20 mM imidazole, 0.5% NP-40, 0.5 mM dithiothreitol (DTT)) supplemented with 1 mM phenylmethylsulphonyl fluoride (PMSF) and 1 × protease inhibitor cocktail (Set IV, CalBiochem). A total of 15 ml of crude lysate was centrifuged at 271,667*g* in a TLA100.3 rotor (Beckman) for 30 min at 4 °C. The cleared supernatant was incubated with 300 μl of Ni-NTA agarose beads (Qiagen), which were subsequently washed with buffer (50 mM HEPES (pH 7.5), 500 mM KCl, 20 mM imidazole, 0.5 mM DTT). Proteins were eluted with 5 × 0.3 ml of elution buffer (50 mM HEPES (pH7.5), 500 mM KCl, 500 mM imidazole, 0.5 mM DTT). Pfy1 was purified from yeast extracts using a poly-L-proline Sepharose column (polyproline affinity chromatography). Recombinant Cof1 and Crn1 (1–600 aa) were purified from bacteria using glutathione *S*-transferase (GST)-affinity chromatography^66^. Protein concentrations were determined using the Gelcode blue staining reagent (Thermo Scientific) with bovine serum albumin as a standard.

Act1 and Act1-120 (E99A and E100A) proteins were purified using DNase-I-based column from haploid WT and *act1-120*, following the protocols described before^67^. In brief, a DNase I affinity column was constructed by conjugating 200 mg of DNase I (Roche, USA) to 10 ml of Affi-Gel (Bio-Rad, USA). An amount of 100 g of frozen yeast were collected, ground by Freezer/Mill and used for actin purification. Clb5–ΔN–Cdk1, Clb3–Cdk1 and Clb2–ΔN–Cdk1 proteins were purified by TAP method as described previously^33^,^68^. In brief, cells carrying a cyclin–Cdk1 complex overexpression cassette under control of the Gal promoter were induced, collected and ground into powder as described above. An amount of 6 g of powder (from ∼2 l of culture) was lysed in lysis buffer (25 mM HEPES-HCl (pH 8.0), 150 mM NaCl, 0.1% NP-40, 1 mM EDTA, 33 mM EGTA, 1 mM PMSF, 1 × protease inhibitor cocktail (Set IV, CalBiochem), 50 mM NaF, 80 mM beta-glycerophosphate, 1 mM Na_3_VO_4_) and centrifuged at 271,667*g* in a TLA100.3 rotor for 30 min at 4 °C. The cleared supernatant was incubated with 200 μl of IgG sepharose beads (GE Healthcare), which were subsequently washed first with IPP150 buffer (25 mM HEPES-HCl (pH 8), 150 mM NaCl, 0.1% NP-40), and then with TEV cleavage buffer (25 mM HEPES-HCl (pH 8), 150 mM NaCl, 0.1% NP-40, 0.5 mM EDTA, 1 mM DTT). Cyclin–Cdk1 complexes were cleaved and eluted by incubation in 1 ml of TEV cleavage buffer and 40 μl of tobacco etch virus (TEV) enzyme at 25 °C for 1 h.

### Reconstitution of actin filament assembly

Reconstitution of actin cable formation from GST-Bni1 FH1-COOH-coated beads was performed essentially as previously described^29^. In brief, hydroxyurea-arrested cells were collected, lysed and resuspended in HK buffer (10 mM HEPES (pH 7.8), 0.1 M KCl) with 1 × protease inhibitors (Protease Inhibitor Cocktail Set IV, Calbiochem, Merck4Biosciences). Cell lysate under the lipid layer was collected and used within 3 h. For actin reconstitution experiments, 1 μl of 2 μm functionalized polystyrene beads (Polysciences, Inc., USA) was added to 19 μl of extract to induce actin cable assembly. A volume of 3 μl of mixture was applied to the surface of a glass slide and covered by a 22 × 22-mm coverslip for imaging.

### Co-immunoprecipitation

Twenty OD_600_ of yeast cells was collected and frozen in liquid N_2_. Cells were washed once with HEN buffer (50 mM HEPES (pH 8.0), 150 mM NaCl, 1 mM EDTA, 1 mM PMSF, 0.5 mM Na_3_VO_4_, 5 mM glycerophosphate, 1 × protease inhibitor cocktail (Set IV, CalBiochem), resuspended in 300 μl of HEN and lysed by glass beads using a Mini-Beadbeater-8 (Bio spec Products. Inc, USA). Crude cell extracts were centrifuged at maximum speed in a microcentrifuge for 10 min at 4 °C. Beads were washed twice with 300 μl of HENN (50 mM HEPES-KOH (pH 8), 150 mM KCl, 1 mM EGTA, 0.1% NP-40), and incubated with 2 μg of Sac6 antibody (lab stock) and Protein G Sepharose 4 FF (GE Healthcare, USA) for 2 h at 4 °C. Protein G Sepharose beads were then washed three times with HENN and subjected to SDS–PAGE.

### Immunoblotting

Yeast whole-cell extracts were prepared as described previously^64^. In brief, cells at OD_600_ of 0.5 in log-phase were collected and washed immediately with 20% trichloroacetic acid (TCA). The cell pellet was suspended in 250 μl of 20% TCA, mixed with an equal volume of glass beads (Sigma, 425–600 μm) and vortexed for 15 min at 4 °C. Glass beads were washed twice with 250 μl of 5% TCA to increase the protein yield. The resulting extract was spun at 13,000 r.p.m. for 5 min at 4 °C. The pellet was dissolved in 100 μl of 2 × SDS-loading buffer (100 mM Tris-HCl (pH 6.8), 4% SDS, 20% glycerol, 0.1% bromophenol blue, 50 mM DTT), neutralized by adding 20 μl of 1 M Tris base, boiled for 5 min and clarified by centrifugation. Total cell extracts were subjected to immunoblot analysis. Proteins were detected using the following primary antibodies: mouse anti-Pgk1 (1:10,000; Invitrogen, 459250), rabbit anti-Clb2 (1:400; Santa Cruz Biotechnology, y-180), rabbit anti-Cof1 (1:2,000), mouse anti-Actin (1:1,000, Thermo Fisher scientific, mAbGEa) and rabbit anti-Sac6 (1:2,000). Blots were subsequently scanned using Odyssey Infrared Imager (LI-COR Biosciences). Full scans are provided in Supplementary Fig. 12.

### Phos-tag acrylamide gels and kinase assays

Phos-tag SDS–PAGE gels to resolve phosphorylated proteins were prepared as previously described^64^. To detect phosphorylation-dependent mobility shifts of Sac6, yeast-purified proteins were loaded onto 8% SDS–polyacrylamide gels containing 20 μM Phos-tag acrylamide (Wako Chemicals USA, Richmond, VA, USA) and 20 μM MnCl_2_, and stained with Gelcode Blue (Thermo Scientific). For kinase assays, to determine the concentrations of the kinases and substrates, both substrates and kinases were subjected to SDS–PAGE along with bovine serum albumin standards and stained with GelCode Blue. The protein concentrations were determined by scanning the image using Image J (http://rsbweb.nih.gov/ij/index.html). A concentration of 20 μM substrate was mixed with 0.1 μM kinase in kinase buffer (50 mM HEPES-KOH (pH 7.5), 100 mM NaCl, 1 mM MgCl_2_, 1 mM DTT), 5 μCi of [γ^32^P] ATP and 200 μM cold ATP. To normalize the kinase activity of different cyclin–Cdk1 complexes, varied amounts of cyclin–Cdk1 complexes in a range from 0.15 to 0.25 μM were mixed with histone H1. Reactions were incubated at room temperature for 20 min and stopped with 4 × SDS-loading buffer.

### Microscopy and image analysis

Yeast strains were grown to log-phase at 25 °C in synthetic media lacking tryptophan, immobilized on concanavalin-A-coated coverslips and then imaged on either Nikon Eclipse Ti-E inverted microscope (Nikon, Tokyo, Japan) or Leica DMi8 (Leica Microsystems, equipped with scientific CMOS (sCMOS) camera as described previously^29^. The actin cables chosen for measuring movement speed were randomly selected from at least 20 cells, in which elongating cables ends could be unambiguously identified. Imaging data were collected using Metamorph software (Molecular Devices) and processed using Image J.

### Actin filament sedimentation

Actin filaments were preassembled for 2 h at 25 °C in F-buffer (10 mM Tris-HCl (pH 7.5), 50 mM KCl, 2 mM MgCl_2_, 1 mM EGTA, 0.2 mM DTT, 0.2 mM ATP, 0.2 mM CaCl_2_). A concentration of 1.25 μM yeast actin filaments were incubated with a range of concentrations of Sac6 protein for 20 min at 25 °C, and then spun at 100,000*g* (high speed) for 20 min at 25 °C in a TLA100 rotor (Beckman). Equal volumes of pellet samples were separated by 12% SDS–PAGE, stained with GelCode Blue and analysed using Image J.

### Mass spectrometry

Mass spectrometry approaches were carried out for phosphorylation site mapping of Sac6 from kinase assays, and for identification and quantification of the Sac6-binding partners by SILAC. Details of sample preparation for Sac6 phosphorylation, SILAC labelling, TAP tag purification and mass spectrometry analysis are described in Supplementary Methods.

### Metadynamics simulation

The peptide in the disordered loop region (from Ala99 to Ile126, APNSTPIVSTAATGLQHKGKGTQAKII) of the Sac6 N terminus with or without phosphorylation on T103 was simulated using the Metadynamic approach (Supplementary Methods, Supplementary Figs 10 and 11).

## Additional information

**How to cite this article:** Miao, Y. *et al*. Fimbrin phosphorylation by metaphase Cdk1 regulates actin cable dynamics in budding yeast. *Nat. Commun.* 7:11265 doi: 10.1038/ncomms11265 (2016).

## Supplementary Material

Supplementary Information Supplementary Figures 1-12, Supplementary Tables 1-3, Supplementary Methods and Supplementary References

Supplementary Data 1 Mass spectrometry data for Sac6 phosphorylation site mapping

Supplementary Data 2 Mass spectrometry data for SILAC coupled to LC-MS/MS. Includes all original data and calculated Light/Heavy ratios for all peptides identified for Sac6 WT (Light)/Sac6-T103A (Heavy).
