# Supplementary material for: Fimbrin phosphorylation by metaphase Cdk1 regulates actin cable dynamics in budding yeast
Source: Nat Commun. 2016 Apr 12;7:11265. doi: 10.1038/ncomms11265 (PMC4832064; doi:10.1038/ncomms11265)
Supplement: Supplementary Information — Supplementary Figures 1-12, Supplementary Tables 1-3, Supplementary Methods and Supplementary References [file ncomms11265-s1.pdf]

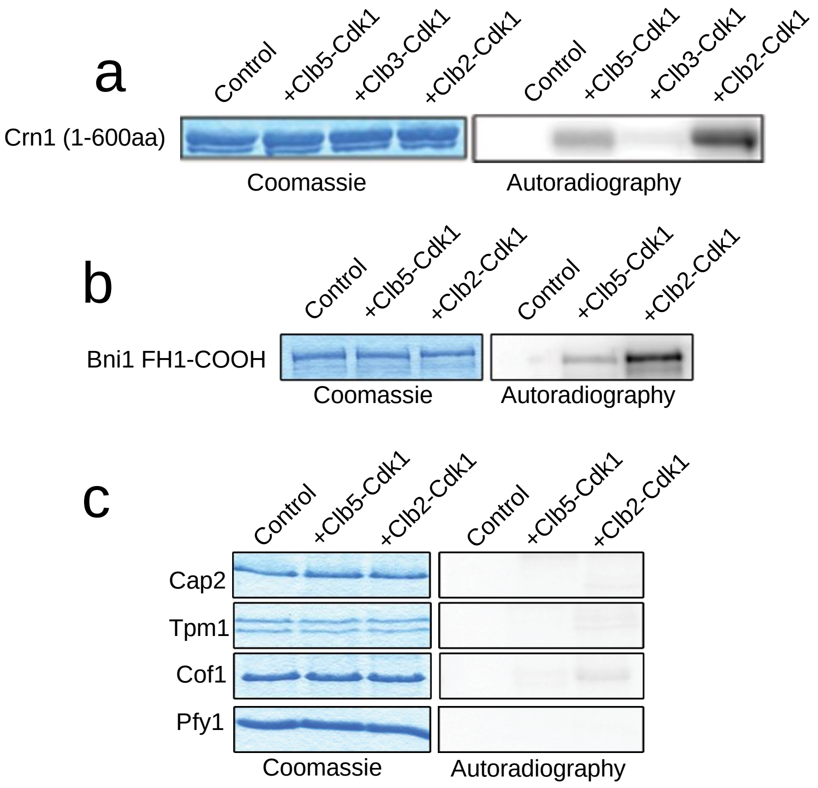

Supplementary Figure 1. Phosphorylation of actin binding proteins by Cdk1.

(a-c) Indicated actin binding proteins Crn1 (1-600aa), Bni1 FH1-COOH, Cap2, Tpm1, Cof1, and Pfy1, were incubated with 6 ng Clb5-ΔN-Cdk1, 3.25 ng of Clb2-ΔN-Cdk1, and/or 5.5 ng of Clb3-Cdk1, respectively, in the presence of [γ32P]-ATP at RT for 20 min. Phosphorylation was analyzed by coomassie blue staining and autoradiography following SDS-PAGE.

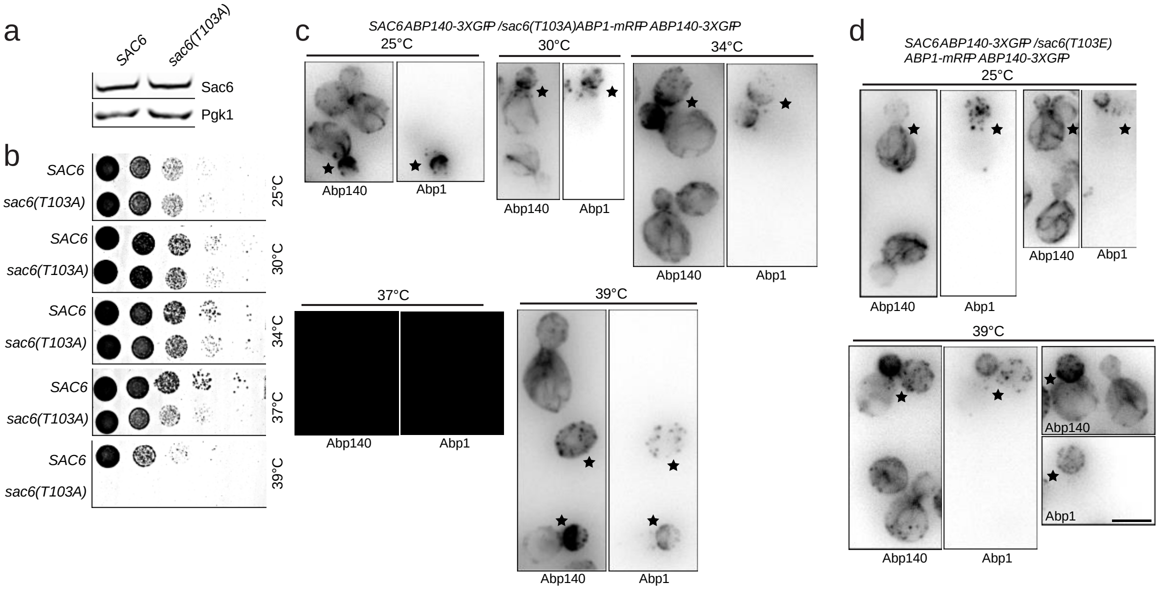

Supplementary Figure 2. Growth and actin cable morphology of *sac6(T103A)* cells. Growth of serially diluted of *SAC6* and *sac6(T103A)* cells on YPD plates. (a) Sac6 expression in *SAC6* and *sac6(T103A)* at 25°C was shown by western blot using antibodies against Sac6 and Pgk1. (b) Plates were incubated at indicated temperature for 24 h. (c, d) Fluorescent pattern of actin filaments in *SAC6*, *sac6(T103A)* (c), or *sac6(T103E)* (d), showed by maximum intensity Z-projection. Cells were pre-incubated for 30 min prior to imaging at indicated temperature. Scale bars, 5 μm.

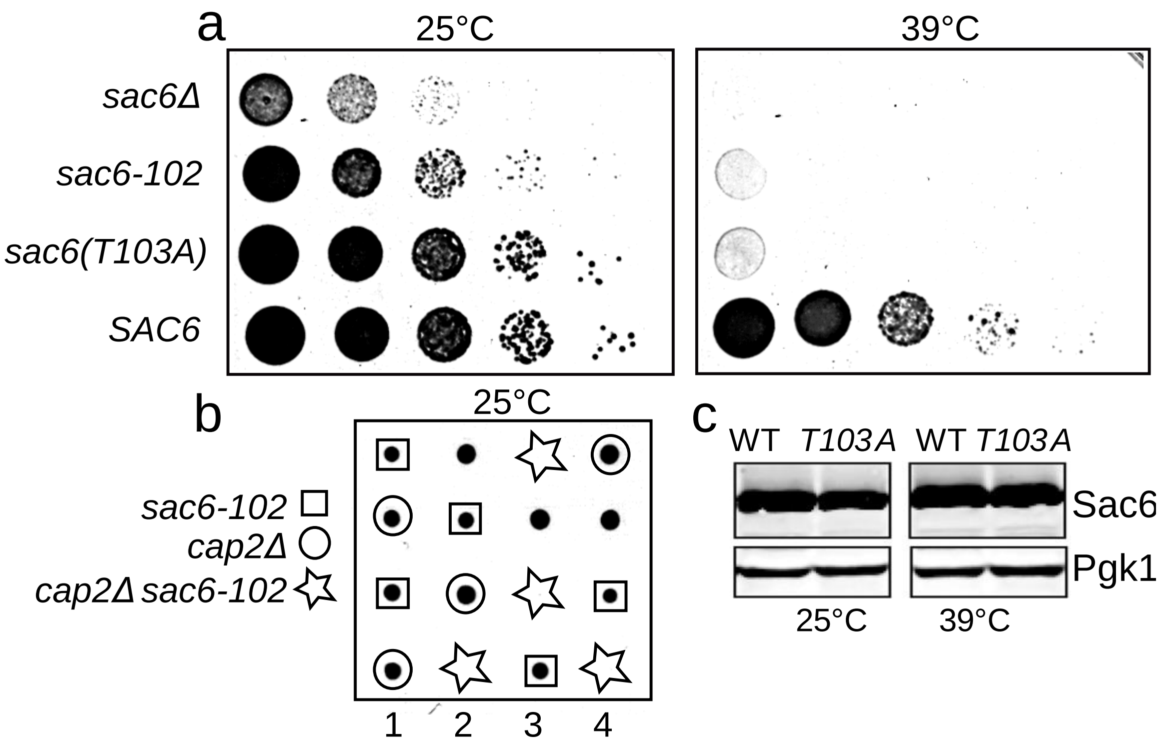

Supplementary Figure 3. Growth and protein expression of *sac6(T103A)* cells. (a) Growth of serially diluted WT, *sac6Δ*, *sac6-102* and *sac6(T103A)* cells on YPD plates. Plates were incubated at indicated temperature for 30 h. (b) Tetrad analysis of heterozygous diploid strains obtained by crossing *cap2Δ* and *sac6-102*. (c) Expression levels of Sac6 and Pgk1 in *SAC6* and *sac6(T103A)* cells grown at 39°C for 30 min prior to immunoblotting.

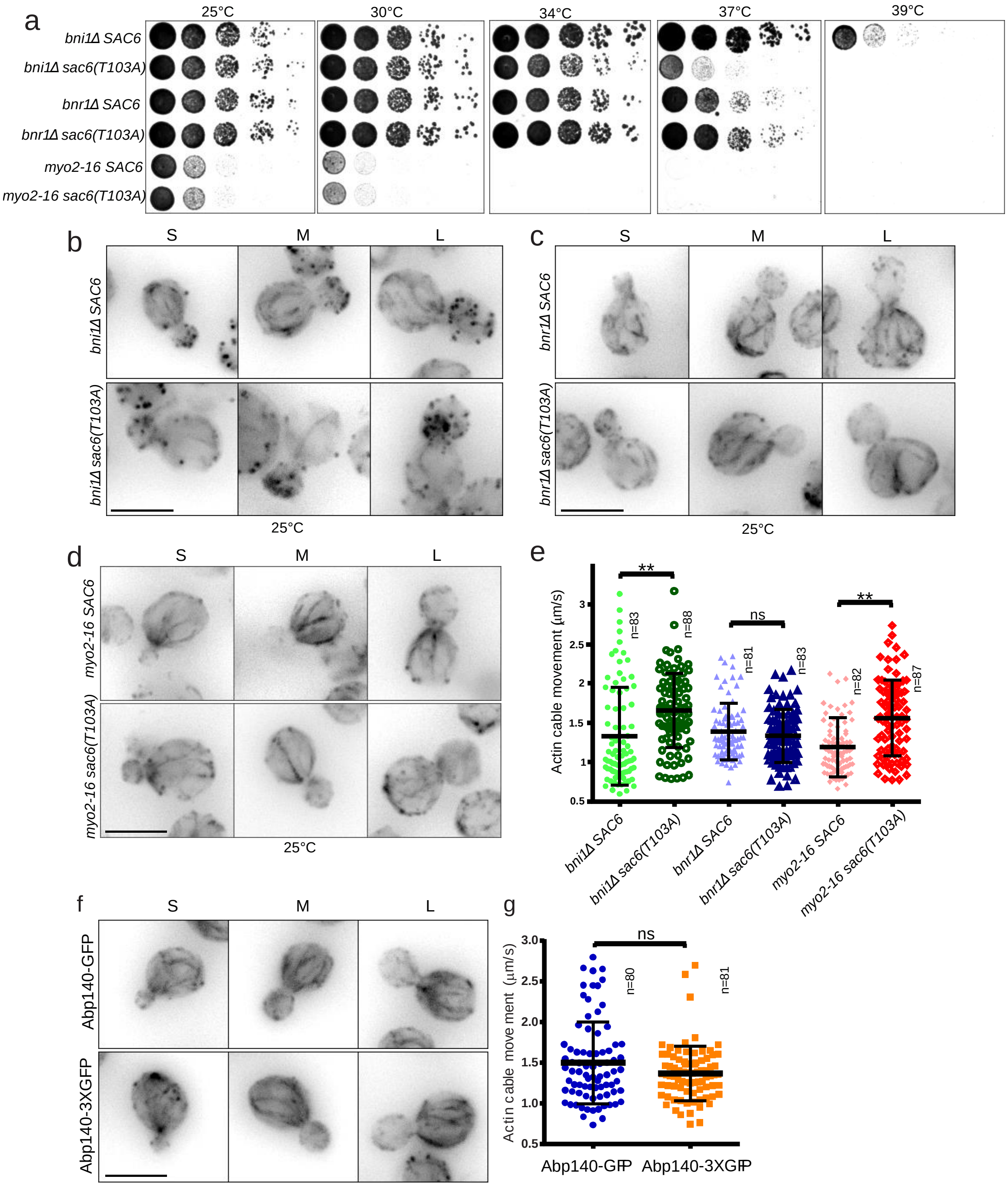
a
25°C
30°C
34°C
37°C
39°C
bni1Δ SAC6
bni1Δ sac6(T103A)
bnr1Δ SAC6
bnr1Δ sac6(T103A)
myo2-16 SAC6
myo2-16 sac6(T103A)
b
S
M
L
bni1Δ SAC6
bni1Δ sac6(T103A)
25°C
c
S
M
L
bnr1Δ SAC6
bnr1Δ sac6(T103A)
25°C
d
S
M
L
myo2-16 SAC6
myo2-16 sac6(T103A)
25°C
e
Actin cable movement (μm/s)
**
ns
**
n=83
n=88
n=81
n=83
n=82
n=87
bni1Δ SAC6
bni1Δ sac6(T103A)
bnr1Δ SAC6
bnr1Δ sac6(T103A)
myo2-16 SAC6
myo2-16 sac6(T103A)
f
S
M
L
Abp140-GFP
Abp140-3XGFP
g
Actin cable move ment (μm/s)
ns
n=80
n=81
Abp140-GFP
Abp140-3XGFP

Supplementary Figure 4. Phospho-regulation of Sac6 affects cell growth and actin cable dynamics in the mutants of formin and type V myosin. (a) Growth of serially diluted *SAC6* and *sac6(T103A)* cells in the background of *bni1Δ, bnr1Δ* and *myo2-16*, respectively, on YPD plates. Plates were incubated at indicated temperature for 33 h. (b-d) Fluorescent pattern of actin cables for *SAC6* and *sac6(T103A)* in indicated mutants at 25°C. S, small-budded cell; M, medium-budded cell; L, large-budded cell. (e) Actin cable movement speed of *SAC6* and *sac6(T103A)* in the background of *bni1Δ*, *bnr1Δ* and *myo2-16*, respectively, at 25°C. (f,g) Actin cable morphology and movement speed of Abp140-GFP and Abp140-3XGFP, respectively. Number of counted actin cables is indicated as "n". ∗∗$p < 0.0001$. ns, no significance. Scale bars, 5 μm.

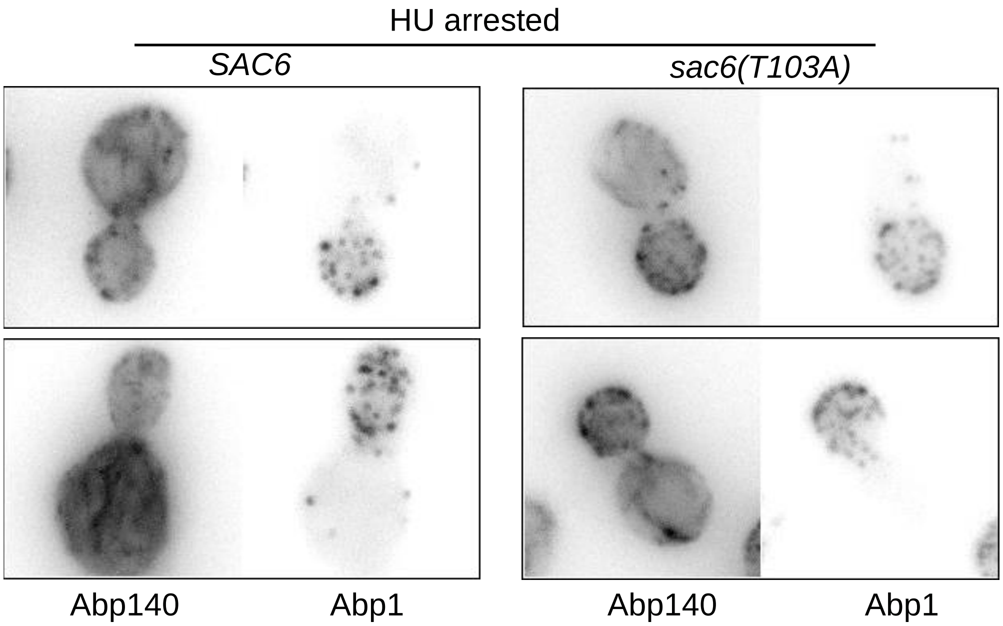

Supplementary Figure 5. Actin cable pattern in HU arrested *SAC6* and *sac6(T103A)* cells. *SAC6* and *sac6(T103A)* cells expressing Abp140-3XGFP and Abp1-mRFP, respectively, were arrested by HU at 25°C. Images were shown by maximum Z-projection.

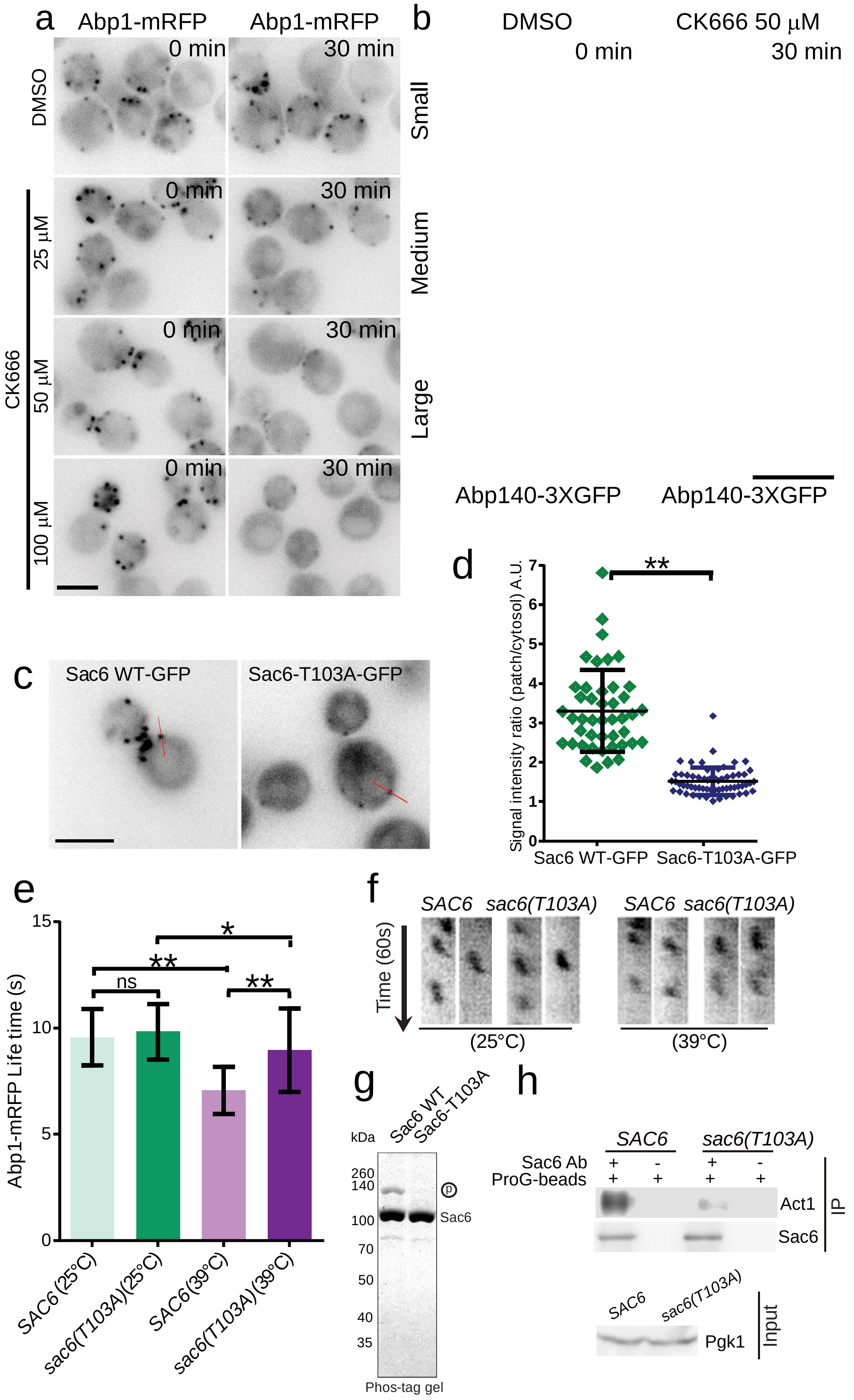
a
Abp1-mRFP
Abp1-mRFP
0 min
30 min
DMSO
CK666
25 µM
50 µM
100 µM
b
DMSO
CK666 50 µM
0 min
30 min
Small
Medium
Large
Abp140-3XGFP
Abp140-3XGFP
c
Sac6 WT-GFP
Sac6-T103A-GFP
d
Signal intensity ratio (patch/cytosol) A.U.
Sac6 WT-GFP
Sac6-T103A-GFP
e
Abp1-mRFP Life time (s)
ns
SAC6(25°C)
sac6(T103A)(25°C)
SAC6(39°C)
sac6(T103A)(39°C)
f
SAC6
sac6(T103A)
Time (60s)
(25°C)
(39°C)
g
kDa
Sac6 WT
Sac6-T103A
Sac6
Phos-tag gel
h
SAC6
sac6(T103A)
Sac6 Ab
ProG-beads
Act1
Sac6
IP
Pgk1
Input

Supplementary Figure 6. Arp2/3 inhibitor dose-response effect on actin structures and Phospho-regulation of Sac6 protein. (a) Abp1-mRFP images of actin patches were acquired in a medial cell focal plane. (b) Z-projection image of actin cables (maximum intensity). (c,d) *In vivo* localisation and distribution of Sac6-GFP and Sac6-T103A-GFP. (e,f) Lifetimes of Abp1-mRFP cortical patches in *SAC6* and *sac6(T103A)* at indicated temperature (n=100). (g) Coomassie blue-stained Phos-tag gels of yeast purified Sac6 WT and Sac6-T103A. (h) Interactions of *SAC6* and *sac6(T103A)* with actin *in vivo* by immunoprecipitation. ∗∗$p < 0.0001$. ∗$p = 0.0002$. ns, no significance. Scale bars, 5 μm.

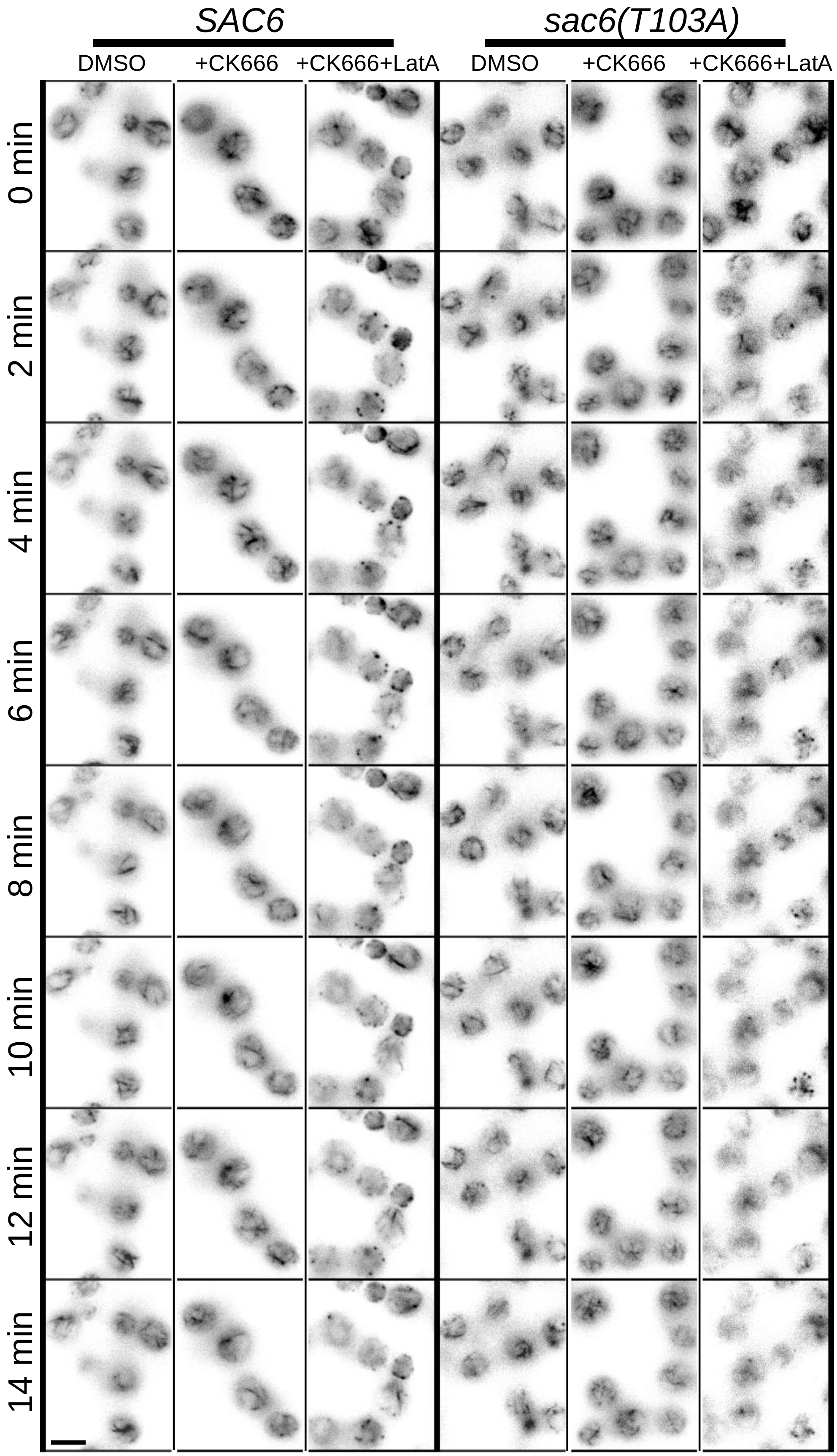
SAC6
sac6(T103A)
DMSO
+CK666
+CK666+LatA
DMSO
+CK666
+CK666+LatA
0 min
2 min
4 min
6 min
8 min
10 min
12 min
14 min

Supplementary Figure 7. Real time imaging of actin cable depolymerisation by Lat A in *SAC6* and *sac6(T103A)* cells. Cells are pretreated with 50 μM CK666 or DMSO for 30 min at RT before adding of 0.4 μM Lat A. Images of actin cables are acquired at the cortex layer of *SAC6* and *sac6(T103A)* cells. Images with 2 min intervals were shown over 14 min period over Lat A application. Scale bars, 5 μm.

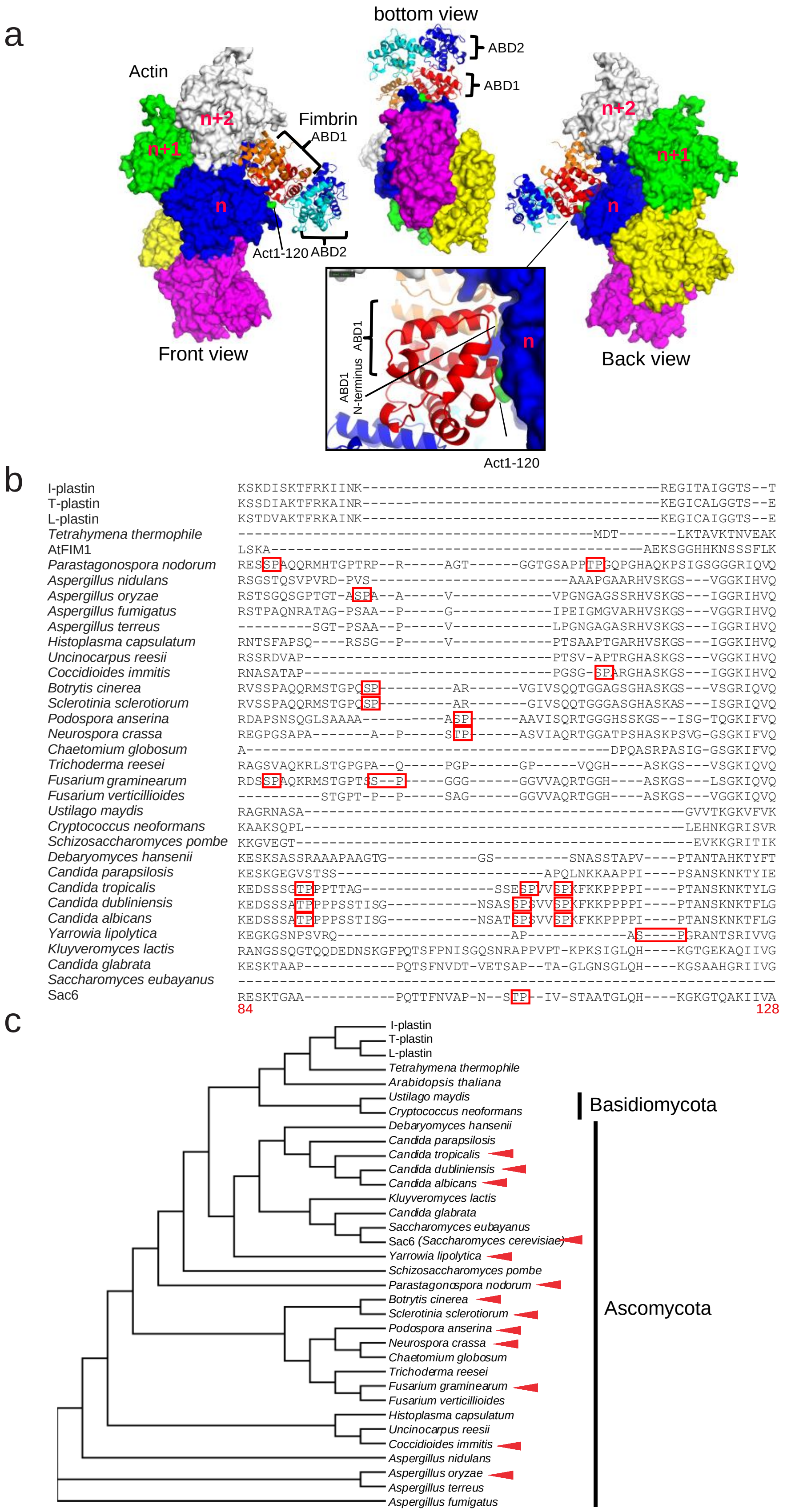

a
bottom view
ABD2
ABD1
Actin
n+2
n+1
n
Fimbrin
ABD1
Act1-120
ABD2
Front view
ABD1
ABD1 N-terminus
n
Act1-120
Back view
n+2
n+1
n
b
I-plastin KSKDISKTFRKIINK-----------------------------------REGITAIGGTS--T
T-plastin KSSDIAKTFRKAINR-----------------------------------KEGICALGGTS--E
L-plastin KSTDVAKTFRKAINK-----------------------------------KEGICAIGGTS--E
Tetrahymena thermophile --MDT-------LKTAVKTNVEAK
AtFIM1 LSKA---AEKSGGHHKNSSSFLK
Parastagonospora nodorum RESSPAQQRMHTGPTRP--R-----AGT------GGTGSAPPTPGQPGHAQKPSIGSGGGRIQVQ
Aspergillus nidulans RSGSTQSVPVRD-PVS------------------------AAAPGAARHVSKGS---VGGKIHVQ
Aspergillus oryzae RSTSGQSGPTGT-ASPA--A-----V------------VPGNGAGSSRHVSKGS---IGGRIHVQ
Aspergillus fumigatus RSTPAQNRATAG-PSAA--P-----G------------IPEIGMGVARHVSKGS---VGGRIHVQ
Aspergillus terreus ---------SGT-PSAA--P-----V------------LPGNGAGASRHVSKGS---IGGRIHVQ
Histoplasma capsulatum RNTSFAPSQ----RSSG--P-----V------------PTSAAPTGARHVSKGS---IGGKIHVQ
Uncinocarpus reesii RSSRDVAP------------------------------PTSV-APTRGHASKGS---VGGKIHVQ
Coccidioides immitis RNASATAP------------------------------PGSG-SPARGHASKGS---IGGKIHVQ
Botrytis cinerea RVSSPAQQRMSTGPQSP---------AR------VGIVSQQTGGAGSGHASKGS---VSGRIQVQ
Sclerotinia sclerotiorum RVSSPAQQRMSTGPQSP---------AR-------GIVSQQTGGGASGHASKAS---ISGRIQVQ
Podospora anserina RDAPSNSQGLSAAAA----------ASP------AAVISQRTGGGHSSKGS--ISG-TQGKIFVQ
Neurospora crassa REGPGSAPA-------A--P-----STP------ASVIAQRTGGATPSHASKPSVG-GSGKIFVQ
Chaetomium globosum A--DPQASRPASIG-GSGKIFVQ
Trichoderma reesei RAGSVAQKRLSTGPGPA--Q-----PGP------GP-----VQGH----ASKGS---VSGKIQVQ
Fusarium graminearum RDSSPAQKRMSTGPTSS--P-----GGG------GGVVAQRTGGH----ASKGS---LSGKIQVQ
Fusarium verticillioides ----------STGPT-P--P-----SAG------GGVVAQRTGGH----ASKGS---VGGKIQVQ
Ustilago maydis RAGRNASA---GVVTKGKVFVK
Cryptococcus neoformans KAAKSQPL---LEHNKGRISVR
Schizosaccharomyces pombe KKGVEGT--EVKKGRITIK
Debaryomyces hansenii KESKSASSRAAAPAAGTG-----------GS---------SNASSTAPV----PTANTAHKTYFT
Candida parapsilosis KESKGEGVSTSS-------------------------APQLNKKAAPPI----PSANSKNKTYIE
Candida tropicalis KEDSSSGTPPPTTAG----------------SSESPVVSPKFKKPPPPI----PTANSKNKTYLG
Candida dubliniensis KEDSSSATPPPPSSTISG-----------NSASSPSVVSPKFKKPPPPI----PTANSKNKTFLG
Candida albicans KEDSSSATPPPPSSTISG-----------NSATSPSVVSPKFKKPPPPI----PTANSKNKTFLG
Yarrowia lipolytica KEGKGSNPSVRQ---------------------AP------------AS----PGRANTSRIVVG
Kluyveromyces lactis RANGSSQGTQQDEDNSKGFPQTSFPNISGQSNRAPPVPT-KPKSIGLQH----KGTGEKAQIIVG
Candida glabrata KESKTAAP-----------PQTSFNVDT-VETSAP--TA-GLGNSGLQH----KGSAAHGRIIVG
Saccharomyces eubayanus ---
Sac6 RESKTGAA-----------PQTTFNVAP-N--STP--IV-STAATGLQH----KGKGTQAKIIVA
84
128
c
I-plastin
T-plastin
L-plastin
Tetrahymena thermophile
Arabidopsis thaliana
Ustilago maydis
Cryptococcus neoformans
Debaryomyces hansenii
Candida parapsilosis
Candida tropicalis
Candida dubliniensis
Candida albicans
Kluyveromyces lactis
Candida glabrata
Saccharomyces eubayanus
Sac6 (Saccharomyces cerevisiae)
Yarrowia lipolytica
Schizosaccharomyces pombe
Parastagonospora nodorum
Botrytis cinerea
Sclerotinia sclerotiorum
Podospora anserina
Neurospora crassa
Chaetomium globosum
Trichoderma reesei
Fusarium graminearum
Fusarium verticillioides
Histoplasma capsulatum
Uncinocarpus reesii
Coccidioides immitis
Aspergillus nidulans
Aspergillus oryzae
Aspergillus terreus
Aspergillus fumigatus
Basidiomycota
Ascomycota

Supplementary Figure 8. Fimbrin docking model and sequence alignment in the disordered region. (a) Multiple vantages of the docking model of an actin filament (PDB ID: 3J8I)[69] with the actin binding core of yeast fimbrin (PDB ID: 1RT8) are shown[70]. Act1-120 (E99A, E100A) mutations are indicated in green on actin monomer “n”. The first two residues on the N-terminus of the yeast fimbrin ABD1 domain (PDB ID: 1RT8) are highlighted in yellow in the magnified view in the box. (b) The presumed disordered region of Sac6 from 84aa to 128aa is aligned with the presumed disordered regions of other fimbrins. Cdk1 substrate consensus sites “SP” and “TP” are highlighted by red boxes. (c) A phylogenic tree of fimbrin proteins in fungi. Fungal species containing consensus Cdk1 sites within N-terminal IDRs of the fimbrin homologs are indicated by red arrows.

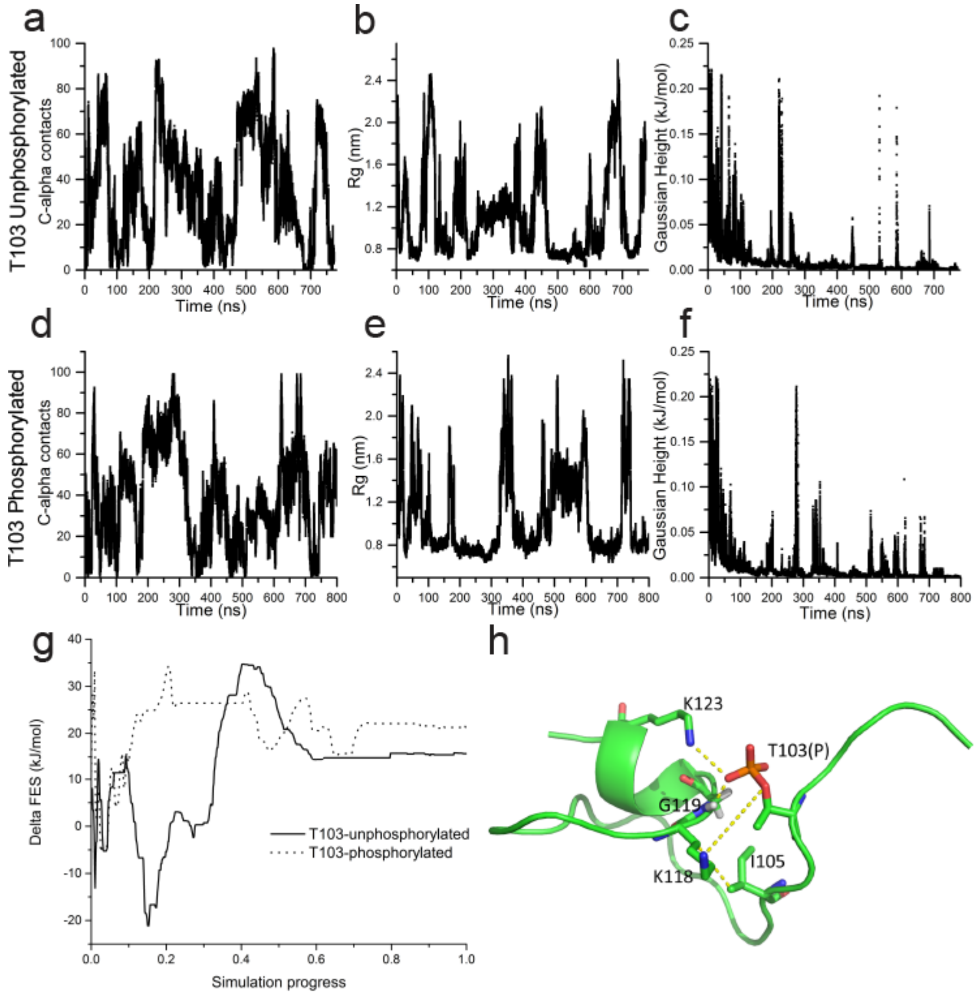

Supplementary Figure 9. Convergence of the metadynamic simulation and side chain interactions of phosphorylated T103. Convergence of the simulations (a-g). The dynamics changes of C-alpha contacts and *Rg* during the simulation for T103 unphosphorylated (a-b) and T103 phosphorylated (d-e) peptides. Two CVs have visited most of the CV space multiple times. (c,f) Gaussian heights approach zero gradually over the simulation. (g) Delta FES (the free energy differences) of the phosphorylated and unphosphorylated T103 peptides. One-dimensional CV (see Supplementary Methods) was used to calculate the free energy differences between two local minima throughout the simulation. Over the simulations, delta FES became stable and its fluctuation was within 1 kJ mol^{-1}. (h) Representative structure of T103 interactions in the lowest energy basin of Figure 4b. Interactions between T103 and K118, G119 and K123 are indicated by yellow dashed lines. The phosphate group of T103 formed a hydrogen bond with the amide proton of G119, and salt bridges with the side chains of K118 and K123.

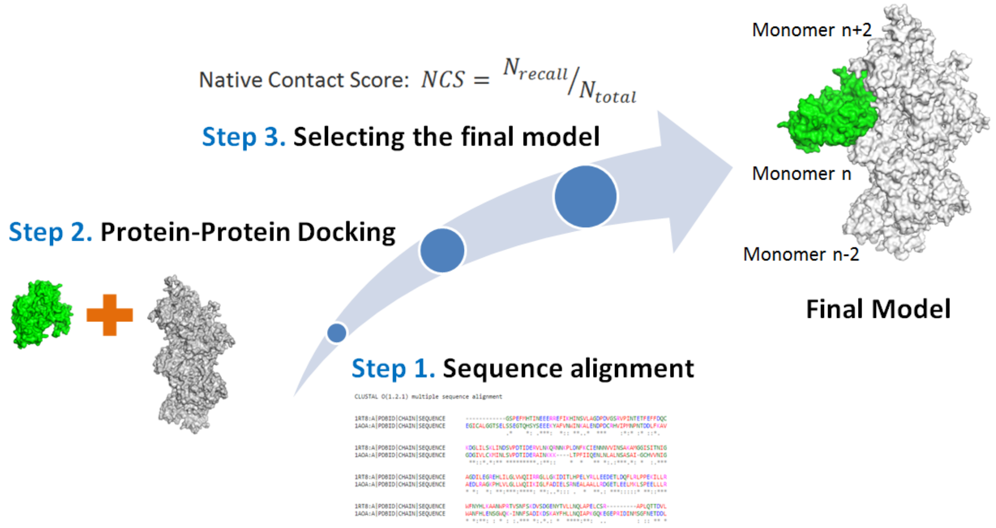

Supplementary Figure 10. The workflow for positioning the yeast fimbrin and F-actin.

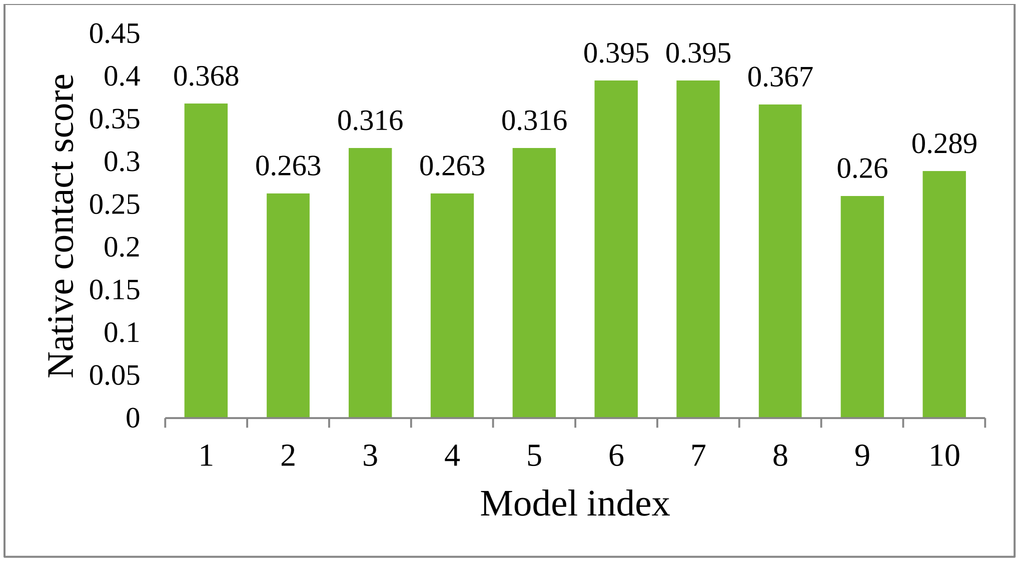

Supplementary Figure 11. The native contact score for the top 10 docking models.

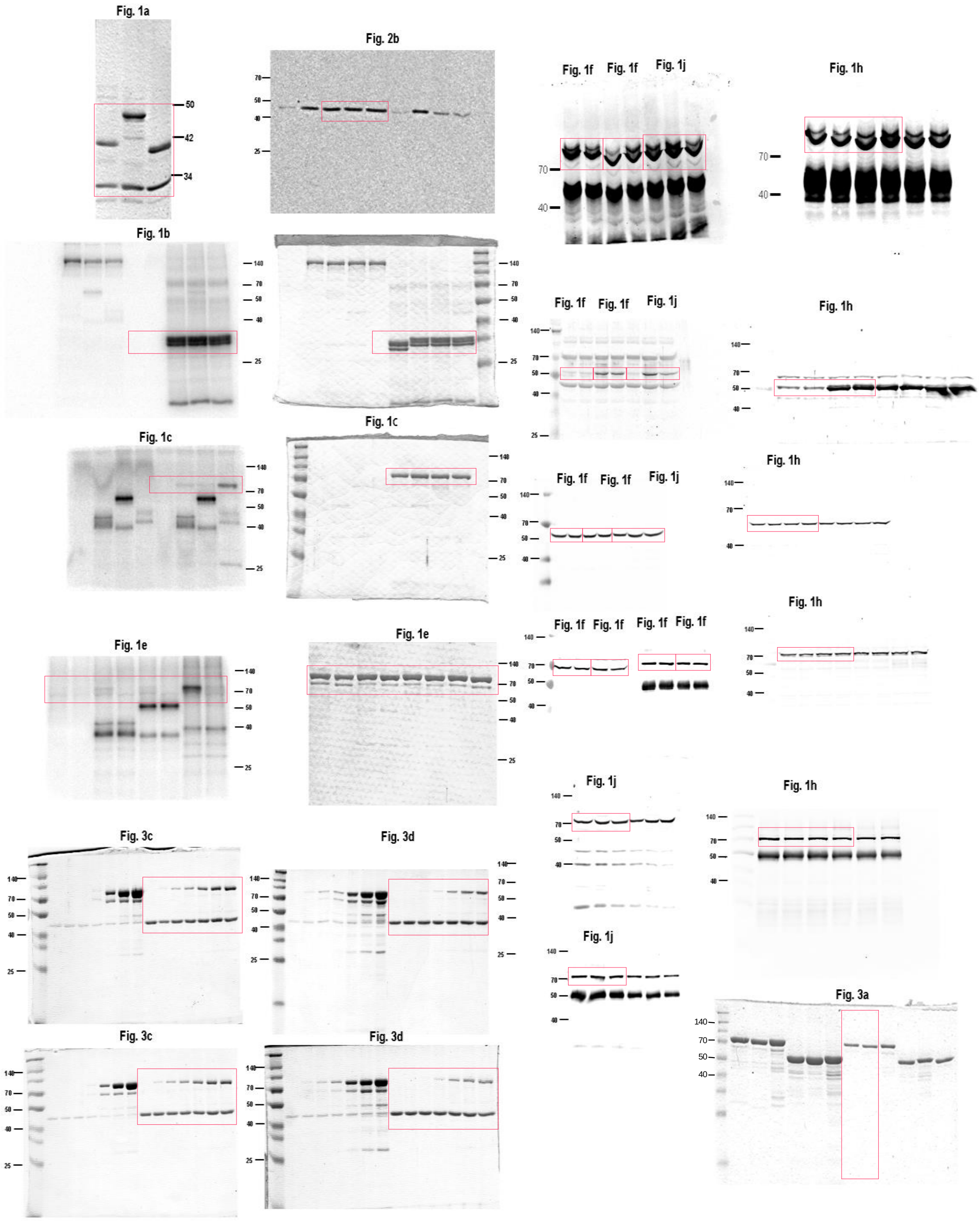

Supplementary Figure 12. Western blot, staining gel, and autoradiography images displayed in the main figures. Red boxes indicate areas were cropped for main figures.

Supplementary Table 1. Yeast strains used in this study.

Strain	Genotype	Note
DDY1102	*MATa/MATα his3-Δ200/his3-Δ200 ura3-52/ura3-52 leu2-3,112/leu2-3,112 lys2-801/LYS2 ade2-1/ADE2*	
DDY904	*MATα his3-Δ200 leu2-3, 112 ura3-52 LYS+*	
DDY4725	*MATa his3-Δ200 leu2-3, 112 ura3-52 sac6Δ::CgLEU2 Abp140-3GFP::HIS3 Abp1-mRFP::HIS3*	This study
DDY4726	*MATa his3-Δ200 leu2-3, 112 ura3-52 sac6Δ::CgLEU2 ura3-52::SAC6::URA3 ABP1-mRFP::HIS3 ABP140-3XGFP::HIS3*	This study
DDY4727	*MATα his3-Δ200 leu2-3, 112 ura3-52 sac6Δ::CgLEU2 ura3-52::sac6(T103A)::URA3 ABP1-mRFP::HIS3 ABP140-3XGFP::HIS3*	This study
DDY4728	*MATα his3-Δ200 leu2-3, 112 ura3-52 sac6Δ::CgLEU2 ura3-52::SAC6::URA3 ABP140-3XGFP::HIS3*	This study
DDY4729	*MATa his3-Δ200 leu2-3, 112 ura3-52 sac6Δ::CgLEU2*	This study
DDY4730	*MATα his3-Δ200 leu2-3, 112 ura3-52 cap2Δ::CgLEU2*	This study
DDY4731	*MATa his3-Δ200 leu2-3, 112 ura3-52 sac6Δ::CgLEU2 ura3-52::SAC6::URA3*	This study
DDY4732	*MATa his3-Δ200 leu2-3, 112 ura3-52 sac6Δ::CgLEU2 ura3-52::sac6(T103A)::URA3*	This study
DDY4733	MATa his3-Δ200 leu2-3, 112 ura3-52 sac6Δ::CgLEU2 ura3-52::sac6(T103A)::URA3 cap2Δ::CgLEU2	This study
DDY4734	*MATα his3-Δ200 leu2-3, 112 ura3-52 sac6Δ::CgLEU2 ura3-52::SAC6::URA3 cap2Δ::CgLEU2 ABP1-mRFP::HIS3 ABP140-3XGFP::HIS3*	This study
DDY4737	*MATa his3-Δ200 leu2-3, 112 ura3-52 sac6Δ::LEU2 ura3-52::SAC6-TAP::URA3::KanMX arg4Δ::NAT lys2-801*	This study
DDY4738	*MATa his3-Δ200 leu2-3, 112 ura3-52 sac6Δ::LEU2 ura3-52::sac6(T103A)-TAP::URA3::KanMX arg4Δ::NAT lys2-801*	This study
DDY4739	*MATa his3-Δ200 leu2-3, 112 ura3-52 SAC6::NAT GAL-CLB2-ΔN::URA3; ADE2 trp1-1(am)::ADH1pr-GAL4BD-hER-VP16:TRP1*	This study
DDY4740	*MATa his3-Δ200 leu2-3, 112 ura3-52 sac6(T103A)::NAT GAL-CLB2-ΔN::URA3; ADE2 trp1-1(am)::ADH1pr-GAL4BD-hER-VP16:TRP1*	This study
DDY4742	*MATα SAC6::NatR can1Δ::STE2pr-Sp_his5 lyp1Δ ura3Δ0 leu2Δ0 his3Δ1 met15Δ0 LYS2*	This study
DDY4743	*MATα sac6(T103A)::NatR can1Δ::STE2pr-Sp_his5 lyp1Δ ura3Δ0 leu2Δ0 his3Δ1 met15Δ0 LYS2*	This study
DDY4744	*MATa ura3-1 trp1-1 leu2-3, 112 his3-11 ade2-1 can1-100 ura3-1::PGAL1-CLB3-TAP-URA3*	Liam Holt
DDY4745	*MATa ura3-1 trp1-1 leu2-3, 112 his3-11 ade2-1 can1-100 ura3-1::PGAL1-CLB2-ΔN-TAP-URA3*	Liam Holt[3]
DDY4746	*MATa ura3-1 trp1-1 leu2-3, 112 his3-11 ade2-1 can1-100 ura3-1::PGAL1-CLB5-ΔN-TAP-URA3*	Liam Holt
DDY4748	*MATα his3-Δ200 leu2-3, 112 ura3-52 sac6-102*	This study
YMY2003	*MATa his3-Δ200 leu2-3, 112 ura3-52 bni1Δ::cgURA3*	This study

	ABP1-mRFP::HIS3 ABP140-3XGFP::HIS3	
YMY2004	*MATa his3-Δ200 leu2-3, 112 ura3-52 bni1Δ::cgURA3 sac6Δ::CgLEU2 ura3-52::sac6(T103A)::URA3 ABP1-mRFP::HIS3 ABP140-3XGFP::HIS3*	This study
YMY2005	*MATa his3-Δ200 leu2-3, 112 ura3-52 bnr1Δ::KanMX sac6Δ::CgLEU2 ura3-52::SAC6::URA3 ABP1-mRFP::HIS3 ABP140-3XGFP::HIS3*	This study
YMY2006	*MATa his3-Δ200 leu2-3, 112 ura3-52 bnr1Δ::KanMX sac6Δ::CgLEU2 ura3-52::sac6(T103A)::URA3 ABP1-mRFP::HIS3 ABP140-3XGFP::HIS3*	This study
YMY2007	*MATa his3-1 leu2-0 met15-0 ura3-0 myo2-16::KanMX*	Boone Lab[4]
YMY2008	*MATa his3-Δ200 leu2-3, 112 ura3-52 myo2-16::KanMX sac6Δ::CgLEU2 ura3-52::sac6(T103A)::URA3*	This study
YMY2009	*MATa his3-Δ200 leu2-3, 112 ura3-52 myo2-16::KanMX ABP1-mRFP::HIS3 ABP140-3XGFP::HIS3*	This study
YMY2010	*MATa his3-Δ200 leu2-3, 112 ura3-52 myo2-16::KanMX sac6Δ::CgLEU2 ura3-52::sac6(T103A)::URA3 ABP1-mRFP::HIS3 ABP140-3XGFP::HIS3*	This study

All strains are in the S288c background except that DDY4744, DDY4745 and DDY4746 are in the W303 background.

Supplementary Table 2. Plasmids used in this study.

Plasmid	Description	Comments
pDD2409	*pGAL-SAC6-3xStreptagII-9xHis*	For purification in yeast
pDD2410	*pGAL-sac6-T103A*-3xStreptagII-9xHis	For purification in yeast

Supplementary Table 3. Primers used in this study.

Primer	Sequence
Sac6-GFP tagging-F	TCGTGCAAGATTAATTATTACTTTTATCGCTTCGTTAATGACTTTGAACAAAcggatccccgggttaattaa
Sac6-GFP tagging-R	ATTGGAACAAGAAAGCTGAGTAGAAAACAGGTTACGAAAGTTGTTTGTTGGCgaattcgagctcgtttaaac
Sac6-knockout-F	ACACTAAAATTATCAGAGAAGAAGCTGATATATTAGCCCTAAGGAGTACACCAAAACACATCGAGGTCGACGGTATC
Sac6-knockout-R	ACGGAGCATTGGAACAAGAAAGCTGAGTAGAAAACAGGTTACGAAAGTTGTTTGTTGGCCGCTCTAGAACTAGTGGATC
Abp140-GFP tagging-F	GTACCGCTGCTGGGTACAAGCTGTGTTTGACGTTCCTCAAcggatccccgggttaattaa
Abp140-GFP tagging-R	TTTATGATGAGAGAGGAGGTGGTACTTGTCTCAGAACTTCgaattcgagctcgtttaaac
Sac6-Gal-OE-F	gggGGATCCATGAATATTGTCAAATTACAAAGAAAATTTCC
Sac6-Gal-OE-R	gggTTAATTAATTTGTTCAAAGTCATTAACGAAGCG
Sac6-pRS306-F;	gggGGATCCTAGTGACTGAAGAAACCGTGGAAC
Sac6-pRS306-R;	gggGAGCTCTATGTGGAAATTGTCAACATGTTTTACAC
Sac6-T103A-F	CAACGTAGCACCTAACTCAGCCCCTATTGTTTCCACTGCTGCCAC
Sac6-T103A-R	GTGGCAGCAGTGGAAACAATAGGGGCTGAGTTAGGTGCTACGTTG
Sac6-T103E-F	CAACGTAGCACCTAACTCAGAACCTATTGTTTCCACTGCTGCCAC
Sac6-T103E-R	GTGGCAGCAGTGGAAACAATAGGTTCTGAGTTAGGTGCTACGTTG

Supplementary Methods

Stable Isotope Labeling by Amino acids in Cell culture (SILAC), extract preparation, and TAP purification. Yeast cell culture and harvesting were done essentially as previously described with some modifications[5]. Yeast cells expressing Sac6 and Sac6-T103A were grown overnight to stationary phase and inoculated (1:10,000) into 1l SILAC labeling SM media with regular, or heavy lysine and arginine, respectively (6.7 g l^{-1} yeast nitrogen base without amino acids, 2% glucose, 2% agar, 5 mg l^{-1} arginine/ L-arginine:HCl (U-^{13}C6, ^{15}N4), 30 mg l^{-1} lysine/ L-lysine:2HCL (^{13}C6, ^{15}N2), 60 mg l^{-1} proline, 200 mg l^{-1} adenine, 10 mg l^{-1} histidine, 60 mg l^{-1} leucine, 10 mg l^{-1} methionine, 20 mg l^{-1} uracil). Cells were grown for at least 10 cell cycles to log-phase (OD_{600} ~0.7). Cells were grown at 39°C for 30 min, harvested by centrifugation for 5 min at 4,000 × rpm at 4°C, washed once with cold H_2O, resuspended in lysis buffer, and immediately lysed for protein extraction by Freezer/Mill. Total soluble protein of each sample was measured by BCA protein assay kit (Pierce Biotechnology, Inc). Cell lysates with equal quantities of proteins from *SAC6-TAP* and *SAC6(T103A)-TAP* were used for TAP tag purifications[3, 6, 7]. IgG-conjugated Dynabeads (M-270 Epoxy beads, Invitrogen, Grand Island, NY) were prepared according to the protocol described[6] and stored at 150 mg ml^{-1} in PBS pH 7.5 plus 0.02% sodium azide at 4°C. 200 µl of IgG-conjugated Dynabeads were washed twice with HKN buffer (50 mM HEPES, pH 8, 150 mM KCl, 0.1% NP-40), and incubated with yeast lysate supernatant that had been centrifuged at 30,000 rpm in a TLA100.3 rotor for 5 min (Beckman). After incubation for 2 h at 4°C, Dynabeads were washed three times with HK buffer and eluted by addition of 200 µl freshly prepared buffer containing 8 M urea in 50 mM Tris-HCl pH 8.8 and incubation for 30 min at RT. About 18 µg protein were eluted from Dynabeads, TCA-precipitated and subjected to MS analysis by MudPIT (Multidimensional Protein Identification Technology).

Mass spectrometry

Sample preparation: The TCA-precipitated protein samples were redissolved in 8 M urea and then split into two aliquots for separate digestions with trypsin and chymotrypsin (Promega). Digestions were carried out at 37 °C overnight at 1:100 enzyme to substrate ratio. Formic acid was added to the combined reactions to a final

concentration of 2%. The peptide mixture was subjected to a TiO2-based phosphopeptide enrichment[8]. Enriched phosphorylated peptide samples were pressure-loaded onto a C18 column (in-house packed, 15 cm x100 um, 5 micron Gemini C18 resin (Phenomenex)). For quantitative analysis, the SILAC labeled proteins were eluted in 8M urea, 50mM Tris, pH 8.8 and then reduced and alkylated with 10 mM Tris(2-carboxyethyl)phosphine hydrochloride (Roche Applied Science) and 55 mM iodoacetamide (Sigma-Aldrich), respectively. The urea was diluted to 2 M with 100 mM Tris pH 8.5 and then the sample was incubated with trypsin overnight at 37 °C (Promega) at a 1:100 enzyme to substrate ratio. The protein digest was pressure-loaded onto a 250 micron inner diameter fused silica capillary (Polymicro Technologies) column with a Kasil frit packed with 3 cm of 5 micron Partisphere strong cation exchange (SCX) resin (Whatman) and 3 cm of 5 micron C18 resin (Phenomenex). After desalting, this bi-phasic column was connected to a 100 micron inner diameter fused silica capillary (Polymicro Technologies) analytical column with a 5 micron pulled-tip, packed with 15 cm of 5 micron C18 resin (Phenomenex) to make a tri-phasic MudPIT column[9].

Mass spectrometry: Each C18 column or MudPIT column was placed in line with an Agilent 1200 quaternary HPLC pump (Agilent Technologies) and the eluted peptides were electrosprayed directly into an LTQ Orbitrap Velos mass spectrometer (Thermo Scientific). The buffer solutions used were 5% acetonitrile/0.1% formic acid (buffer A), 80% acetonitrile/0.1% formic acid (buffer B) and 500 mM ammonium acetate/5% acetonitrile/0.1% formic acid (buffer C). For phosphopeptide analysis, a 150 min gradient from 10% to 50% acetonitrile in 0.1% formic acid was used with a flow rate of ~300 nl min^{-1} (through split). For SILAC quantification analysis, a 12-step MudPIT was run with salt pulses of 0, 10, 20, 30, 40, 50, 60, 70, 80 and 100% buffer C and 90% buffer C/10% buffer B (twice). In each MudPIT step, the 120 min elution gradient had the following profile: 10% buffer B beginning at 15 min to 45% buffer B at 105 min. MS instrument method consisted of one FT full-scan mass analysis (300-1600 m/z, AGC = 1E6, 120000 resolving power at m/z = 400) followed by 20 data-dependent collision induced dissociation (CID) MS/MS spectra (AGC = 1E4, 2 m/z isolation window, normalized collision energy at 35%) with dynamic exclusion for 120 s. Application of mass

spectrometer scan functions and HPLC solvent gradients were controlled by the Xcalibur data system (Thermo Scientific).

Mass spectrometry data analysis: MS/MS spectra were extracted using RawXtract (version 1.9.9)[10] and searched with the ProLuCID algorithm (http://fields.scripps.edu/prolucid/) against a *Saccharomyces cerevisiae* database concatenated to a decoy database in which the sequence for each entry in the original database was reversed[11]. For phosphopeptide analysis, the ProLuCID search was performed using differential modification of serine and threonine due to phosphorylation (79.9663). No enzymatic cleavage conditions were imposed on the database search. For protein identifications, the "light" ProLuCID search was performed using no enzyme specificity and static modification of cysteine due to carboxyamidomethylation (57.02146); the "heavy" ProLuCID search was performed and additionally considered static modification of [$^{13}C_6$ $^{15}N_4$] arginine (10.0083) and [$^{13}C_6$ $^{15}N_2$] lysine (8.0142). The search results were assembled and filtered using the DTASelect (version 2.0) algorithm[12]. For protein identification, the protein false positive rate was kept below one percent and the average mass deviation was less than 5 ppm. For phosphopeptide identification, only modified peptides were considered and the peptide false positive rate was kept below one percent. For SILAC analysis, the ratio of a binding partner for Sac6 and to Sac6-T103A was calculated with the extracted liquid chromatography peak area for each peptide and the ratio determined with linear regression. To focus on the bona fide interaction proteins, we discarded the protein hits with less than two peptides. We also set up a cutoff for proteins with Sac6 to Sac6-T103 ratio $\leq$0.5 and $\geq$2 to remove the partners that bind to Sac6 and Sac6-T103A equally, as well the nonspecific hits that are pulled-down with the affinity matrix[13].

Metadynamics simulation. We simulated the peptides, with or without phosphorylation on T103 (from Ala99 to Ile126), starting from their linear status. Acetyl and Amine protection groups were used at the N and C termini, respectively (Acetyl-APNSTPIVSTAATGLQHKGKGTQAKII-Amine). The peptides were simulated in TIP3P water environment with NaCl at 150 mM in periodic boundary conditions[14].

Amber99sb-ildn force field was applied for the unphosphorylated amino acids[15], while the force field parameters phosphorylated threonine were adopted from R. A. Bryce' amber parameter database[16]. With energy minimization and NPT equilibration, both T103 phosphorylated and unphosphorylated peptides were subjected to short molecular dynamics using Gromacs 4.6.5 package[17] and followed by metadynamics approach by Plumed 2.1[18] patched with Gromacs 4.6.7. The velocity rescaling thermostat with a time constant 0.1 ps was used. The water molecules in simulation boxes are 11922 and 12719 for T103-unphosphorylated and T103-phosphorylated simulation systems, respectively. The box sizes are 375.1 nm^3 and 401.9 nm^3, respectively, for T103-unphosphorylated and T103-phosphorylated system, which are large enough to prevent the contact between periodic images when the peptide is in extended conformation. All the bonds between heavy atoms and hydrogen atoms were fixed according to SHAKE algorithm[19], while the LINCS algorithm[20] was applied to restrict the covalent bonds between heavy atoms to an equilibrium value. Long range electrostatic interaction was treated by PME scheme[21]. 1.0 nm and 1.2 nm were used as the cutoff for long range electrostatic interaction and van der Waals.

1000 steps of energy minimisation was performed and followed by a 100 ps NPT equilibration with fixed heavy atoms and free water molecules and ions. Then a 10 ns NVT molecular dynamics simulation under 300 K was performed. The time step was set as 2 fs. The last frame structure from the 10 ns NVT simulation was adopted as the initial structure for the well-tempered metadynamics simulation[22, 23]. Two collective variables (CVs), were adopted. CV creates an approach to monitor the simulation system in a manner by simplifying the large number of parameters that are contained in simulation system. The first CV is the radius of gyration (*Rg*) of all the heavy atoms of the peptide, which calculates the mass averaged distance of all atoms to the center of the peptide. More extended or linear peptides produce larger *Rg*. The second CV is the alpha carbon atom contact number, which concerns the total number of the pairwise alpha carbon atoms (in each amino acids) contacts between residue k and residue >= k+5 (k is the residue index number). If the distance between a pair of C alpha atoms is less than 0.85 nm, it was counted as one contact number. Because the CVs need to be continuous for simulation, the C-alpha contact number was transformed by a rational switching function:

$$s(r) = \sum \frac{1-(\frac{r-r_0}{r_0})^n}{1-(\frac{r-r_0}{r_0})^m}$$

where *s(r)* is the contact number value, while r is the instantaneous distance, r0 is the cutoff distance (0.85 nm), m and n were set as 12 and 6. A greater C-alpha contact number indicates more internal interactions and thus a more compact level in folding. For the metadynamics simulations, the bias factor was set to 10, while the Gaussian height was 0.2 kJ mol^{-1} and weight parameters were 1.0 and 0.1 nm for *C-alpha contacts* and *Rg* respectively. The Gaussian deposit stride was 1 ps. After completion of the simulation, the convergence of the metadynamics simulations of both peptides was verified. The Delta FES (free energy difference) between two local minima along one-dimensional CV (*C-alpha contacts* for T103-unphosphorylated peptide and *Rg* of T103-phosphorylated peptide) was plotted against the progress of simulation. The approach for calculating Delta FES was adopted from Plumed 2.1 (http://plumed.github.io/doc-v2.2/user-doc/html/belfast-6.html). Toward the end of simulation, though the diffusive behaviors of the CVs were overserved, the free energy difference between two local minima remains stable (less than 1 kJ mol^{-1}), indicating the convergence.

The free energy surface of the peptides plotted against two variables (*C-alpha contacts* and *Rg*) was calculated by Plumed 2.1[23]. In addition, the trajectories produced by metadynamics simulations were used for clustering analysis by Gromacs g_cluster every 50 ps. The Gromos method and cutoff of 0.3 nm were used for clustering. The clustering analysis groups structures based on pair-wise RMSD. Afterwards, frames of the trajectories were assigned a cluster index number. The cluster index information and bias potential were fed to reweighting analysis[24] to obtain the unbiased free energies. The center structures of the three smallest free energy clusters were extracted and displayed using PyMol 1.3.

To create the contact probability map, we first mapped minimum distance contact between residue side chains for Thr103-unphosphorylated and phosphorylated peptides, respectively. Total 1159 and 1852 structures in lowest energy basin of Thr103-unphosphorylated (*Rg*, 0.9-1.1; *C-alpha contacts*, 45.0-55.0) and Thr103 phosphorylated peptides (*Rg*, 0.9-1.1; *C-alpha contacts*, 35.0-45.0) were used for generating Fig. 4a,b.

For our simulated T103 containing peptides, contact probability map of 27×27 bins (lattices) for each structure was constructed concerning the distances for each pair of residues. If any side chain heavy atom of one residue locates within a distance <0.6 nm of any other residue atom, a score of 1.0 was given. Then, the average score of each lattice was assigned for the final map. Half of each symmetric map for Thr103-unphosphorylated and -phosphorylated peptides was combined to form a final contact probability map.

Modeling of actin binding core of fimbrin (ABD1 and ABD2) associated with an actin filament.

Neither a crystal structure nor a Cryo-EM structure for the actin crosslinking cores of budding yeast fimbrin (ABD1 and ABD2) docked to F-actin is currently available. To position both the ABD1 and ABD2 of yeast fimbrin on an actin filament in an atomic model we used the protein-protein docking approach ClusPro (http://cluspro.bu.edu/login.php) in Figure 3b and supplementary Figure 8a. The currently available actin crosslinking core of *S. pombe* fimbrin (PDB ID: 1RT8)[2] and the highest resolution Cryo-EM structure of an actin filament (PDB ID: 3J8I)[1] were used to construct the final model. Docking of 1RT8 (ABD1 + ABD2) and 3J8I was based on a previously reported model of T-fimbrin ABD1 with F-actin[25]. The workflow for modeling is shown in Supplementary Figure 10.

Step 1, The structure reported by Hanein, et al[25] provided the position and binding score for residues at the interface between ABD1 of T-fimbrin and F-actin. To position 1RT8 (ABD1 + ABD2) on F-actin, we first identified the residues of the ABD1 of 1RT8 that interact with F-actin by aligning ABD1 of *S. pombe* fimbrin (1RT8, residue index 121-375) with ABD1 of T-fimbrin (1AOA, residue index 110-380) using EMBI Clustal Omega (http://www.ebi.ac.uk/Tools/msa/clustalo/).

Step 2. The binding interface for 1RT8 and 3J8I was predicted using the ClusPro Protein-Protein docking server (http://cluspro.bu.edu/home.php)[26, 27]. F-actin (PDB ID: 3J8I) and *S. pombe* fimbrin (PDB ID: 1RT8) were submitted as receptor and ligand, respectively. To facilitate precise docking, the deduced position of 1RT8 residues by step 1, which have a corresponding binding score higher than 0.9[25], were submitted as

“attractive residue”. Upon completion of the docking by ClusPro, the top 10 ranked models were extracted (Supplementary Figure 11; 1-10).

Step 3. To choose the best model, we wrote python scripts to find the interacting residues (with heavy atom distances of less than 4 Angstroms) in each model, and then evaluated the native contact scores the top 10 models derived from ClusPro. The native contact score is defined by the ratio of N_{recall}/N_{total} (Supplementary Figure 10). N_{recall} is the number of actin residues generated by ClusPro that match the reported actin residues facing T-fimbrin[25]. N_{total} (= 36) is the total number of original reported actin residues[25]. The higher N_{recall}/N_{total} score reflects a better conformation and position model for the complex of *S. pombe* fimbrin and F-actin. We found that both model 6 and 7 have the highest score, 0.395. Then interface residues in both models were further examined. We found that ABD1 interacts with actin monomer “*n*” and “*n-2*” in model 7 but interacts with “*n*” and “*n+2*” in model 6, which is consistent with the reported conformation for the complex between ABD1 and F-actin[25]. Therefore, model 6 was used to define the conformation and position of ABD1 and ABD2 of the *S. pombe* fimbrin and F-actin binding complex.

Supplementary References

1. Galkin VE, Orlova A, Vos MR, Schroder GF, Egelman EH. Near-atomic resolution for one state of f-actin. *Structure* **23**, 173-182 (2015).

2. Klein MG, *et al.* Structure of the actin crosslinking core of fimbrin. *Structure* **12**, 999-1013 (2004).

3. Holt LJ, Tuch BB, Villen J, Johnson AD, Gygi SP, Morgan DO. Global analysis of Cdk1 substrate phosphorylation sites provides insights into evolution. *Science* **325**, 1682-1686 (2009).

4. Li Z, *et al.* Systematic exploration of essential yeast gene function with temperature-sensitive mutants. *Nat Biotechnol* **29**, 361-367 (2011).

5. de Godoy LM, Olsen JV, de Souza GA, Li G, Mortensen P, Mann M. Status of complete proteome analysis by mass spectrometry: SILAC labeled yeast as a model system. *Genome Biol* **7**, R50 (2006).

6. Alber F, *et al.* Determining the architectures of macromolecular assemblies. *Nature* **450**, 683-694 (2007).

7. Alber F, *et al.* The molecular architecture of the nuclear pore complex. *Nature* **450**, 695-701 (2007).

8. Cantin GT, Shock TR, Park SK, Madhani HD, Yates JR, 3rd. Optimizing TiO2-based phosphopeptide enrichment for automated multidimensional liquid chromatography coupled to tandem mass spectrometry. *Anal Chem* **79**, 4666-4673 (2007).

9. Washburn MP, Wolters D, Yates JR, 3rd. Large-scale analysis of the yeast proteome by multidimensional protein identification technology. *Nat Biotechnol* **19**, 242-247 (2001).

10. McDonald WH, *et al.* MS1, MS2, and SQT-three unified, compact, and easily parsed file formats for the storage of shotgun proteomic spectra and identifications. *Rapid communications in mass spectrometry : RCM* **18**, 2162-2168 (2004).

11. Peng J, Elias JE, Thoreen CC, Licklider LJ, Gygi SP. Evaluation of multidimensional chromatography coupled with tandem mass spectrometry (LC/LC-MS/MS) for large-scale protein analysis: the yeast proteome. *J Proteome Res* **2**, 43-50 (2003).

12. Tabb DL, McDonald WH, Yates JR, 3rd. DTASelect and Contrast: tools for assembling and comparing protein identifications from shotgun proteomics. *J Proteome Res* **1**, 21-26 (2002).

13. Trinkle-Mulcahy L, *et al.* Identifying specific protein interaction partners using quantitative mass spectrometry and bead proteomes. *J Cell Biol* **183**, 223-239 (2008).

14. Jorgensen WL, Chandrasekhar J, Madura JD, Impey RW, Klein ML. Comparison of simple potential functions for simulating liquid water. *The Journal of chemical physics* **79**, 926-935 (1983).

15. Lindorff-Larsen K, *et al.* Improved side-chain torsion potentials for the Amber ff99SB protein force field. *Proteins* **78**, 1950-1958 (2010).

16. Piana S, Lindorff-Larsen K, Shaw DE. How robust are protein folding simulations with respect to force field parameterization? *Biophysical journal* **100**, L47-L49 (2011).

17. Pronk S, *et al.* GROMACS 4.5: a high-throughput and highly parallel open source molecular simulation toolkit. *Bioinformatics*, btt055 (2013).

18. Bonomi M, *et al.* PLUMED: A portable plugin for free-energy calculations with molecular dynamics. *Computer Physics Communications* **180**, 1961-1972 (2009).

19. Ryckaert J-P, Ciccotti G, Berendsen HJ. Numerical integration of the cartesian equations of motion of a system with constraints: molecular dynamics of n-alkanes. *Journal of Computational Physics* **23**, 327-341 (1977).

20. Hess B, Bekker H, Berendsen HJ, Fraaije JG. LINCS: a linear constraint solver for molecular simulations. *Journal of computational chemistry* **18**, 1463-1472 (1997).

21. Darden T, York D, Pedersen L. Particle mesh Ewald: An N· log (N) method for Ewald sums in large systems. *The Journal of chemical physics* **98**, 10089-10092 (1993).

22. Barducci A, Bussi G, Parrinello M. Well-tempered metadynamics: a smoothly converging and tunable free-energy method. *Physical review letters* **100**, 020603 (2008).

23. Bonomi M, Barducci A, Parrinello M. Reconstructing the equilibrium Boltzmann distribution from well-tempered metadynamics. *Journal of computational chemistry* **30**, 1615-1621 (2009).

24. Tiwary P, Parrinello M. A time-independent free energy estimator for metadynamics. *J Phys Chem B* **119**, 736-742 (2015).

25. Hanein D, *et al.* An atomic model of fimbrin binding to F-actin and its implications for filament crosslinking and regulation. *Nature structural biology* **5**, 787-792 (1998).

26. Kozakov D, *et al.* How good is automated protein docking? *Proteins* **81**, 2159-2166 (2013).

27. Kozakov D, Brenke R, Comeau SR, Vajda S. PIPER: an FFT-based protein docking program with pairwise potentials. *Proteins* **65**, 392-406 (2006).
